# Supplementary material for: Harnessing screw dislocations in shell-lattice metamaterials for efficient, stable electrocatalysts
Source: Nat Commun. 2025 Aug 7;16:7273. doi: 10.1038/s41467-025-62489-0 (PMC12332154; doi:10.1038/s41467-025-62489-0)
Supplement: Supplementary file 1 — Supplementary Information [file 41467_2025_62489_MOESM1_ESM.pdf]

Supplementary Information

Harnessing screw dislocations in shell-lattice metamaterials for efficient, stable electrocatalysts

Liqiang Wang [1, †], Di Yin [2, †], James Utama Surjadi [3, †], Junhao Ding [4], Huangliu Fu [5], Xin Zhou [6], Rui Li [4], Mengxue Chen [7], Xinxin Li [8], Xu Song [4, *], Johnny C. Ho [2, *], Yang Lu [8, *]

1 Department of Mechanical Engineering, City University of Hong Kong, Hong Kong, China
2 Department of Materials Science and Engineering, City University of Hong Kong, Kowloon, China
3 Department of Mechanical Engineering, Massachusetts Institute of Technology, Cambridge, MA, USA
4 Department of Mechanical and Automation Engineering, The Chinese University of Hong Kong, Hong Kong, China
5 Shenyang National Laboratory for Materials Science, Institute of Metal Research, Chinese Academy of Sciences, Shenyang, China
6 Physikalisches Institut, Westfälische Wilhelms-Universität, Münster 48149, Germany
7 Department of Chemistry, City University of Hong Kong, Hong Kong, China
8 Department of Mechanical Engineering, The University of Hong Kong, Hong Kong, China

† These authors contributed equally: Liqiang Wang, Di Yin, and James Utama Surjadi
* Corresponding authors: ylu1@hku.hk, johnnyho@cityu.edu.hk, and xsong@mae.cuhk.edu.hk.

This supplementary information file contains the following:

Supplementary Notes (Note 1-5)
Supplementary Methods (Method 1-4)
Supplementary Table (Table S1-3)
Supplementary Figures (Figure S1-44)

Other supplementary information files contain:

Supplementary Movie 1

Supplementary Note 1:

The specific roles of each step in the development of the metamaterial catalyst

DLP 3D printing:

We use DLP technology to 3D printing the organogel scaffolds and then soaked in deionized water for 6 h at room temperature to convert to hydrogel scaffolds. This step mainly aims to create highly designable 3D architectures as the support of the catalysts, which determines the macroscale topology morphology of final catalytic electrodes.

Metal salt precursor immersion:

The hydrogel scaffolds are immersed in a corresponding metal salt precursor solution, allowing Fe-Co-Ni ions to infuse into the architecture. The precursor concentration of metal salt solution in this step plays a crucial role in introducing screw dislocations during the subsequent sintering and reduction process. First, we will provide some background on screw dislocation-driven nanomaterial growth. Since most catalytic nanostructures are designed to be anisotropic to maximize the exposure of electrochemically active sites, their formation process is fundamentally governed by the mechanisms of crystal growth[1]. The challenge to grow anisotropic nanostructures is to break the symmetry in crystal growth. In many studies, the use of catalysts or templates is a widely employed approach to regulate crystal growth and achieve anisotropic nanostructures [2,3]. Screw dislocation mechanism is another strategy to synthesize anisotropic nanomaterials. According to the classical crystal growth theory, the supersaturation (σ) works as the driving force for crystal growth[4].

$$\sigma = \ln\left(\frac{c}{c_0}\right) \quad (1)$$

where c and c_0 are the precursor concentration and equilibrium concentration of system. At low supersaturation conditions, screw dislocation defects can promote the crystal growth. The line of a screw dislocation creates step edges upon intersection with a crystal surface, which will propagate as self-perpetuating growth spirals. As supersaturation is increased, growth mode will transfer to layer-by-layer (LBL) growth and dendritic growth progressively[5] (Supplementary Fig. 3b). Therefore, through intentionally exploiting low supersaturation conditions, many researchers achieved catalyst-free synthesis of a variety of anisotropic nanomaterials, including nanowires, nanoplates, and 3D hyperbranching nanostructures. Based on this theory, by utilizing low concentrations of Fe-Co-Ni metal salt solutions (2M), we have, for the first time, integrated this approach into the 3D printing process to *in situ* create helical nanoplates on the surface of the architecture. This step can be considered as the bridge between macroscale architecture fabrication and nanoscale nanomaterials synthesis.

Calcination and reduction:

The metal-salt-rich hydrogel scaffolds were calcinated in air to remove the polymer and convert to metal oxides. From the SEM image (Supplementary Fig. 3a), we observed that Fe-Co-Ni oxides exist in the form of nanoparticles and are clustered together. Finally, the metallic oxide nanoparticles were sintered, reduced, and transformed into a single solid-solution structure. This step can be considered as the final visualization process, where the metallic 3D framework is formed while surface nanostructures are simultaneously grown on the architecture, directly determining the morphology of interface-free catalyst. The morphology feature of nanomaterial was considered to

mainly depend on screw dislocation growth kinetics. At a low supersaturation, when growth velocities and directions of steps at the dislocation core (V_c) is equal to those at the outer edges (V_0), the newly generated steps near the dislocation core propagate at the same rate with earlier steps at the outer edge of the growth spiral[6], inducing the growth of the nanoplates (Supplementary Fig. 3c). Since the growth of the architecture and surface nanoplates is completed simultaneously during the reduction process, leading to the formation of an interface-free feature between the support and surface nanomaterials.

Supplementary Note 2:

Modeling dual-scale shell-lattice metamaterials

Firstly, to model the TPMS structure, we employed an approach to extract the zero iso-surface of the generalized equation based on an implicit function [7]:

$$f(X,Y,Z) = c \quad (2)$$

$$where\ X = \frac{2\pi}{L}x; Y = \frac{2\pi}{L}y;\ Z = \frac{2\pi}{L}z \quad (3)$$

Where X, Y, and Z indicate the period in different directions, L defines the size of the unit cell, C as a constant value determines the volume fraction of the structure. One-scale gyroid sheet-network structure (G type TPMS), where the solid sheet is enclosed by two surfaces with different C, can be generated by the following implicit functions:

$$f_G(X,Y,Z) = \cos(X)\sin(Y) + \cos(Y)\sin(Z) + \cos(Z)\sin(X) = c \quad (4)$$

Dual-scale structures can be constructed via Boolean operations between two models with single scale.

$$Intersection\text{: } \emptyset\_(A \cap B)(X,Y,Z) = \max(\emptyset\_A(X,Y,Z), \emptyset\_B(X,Y,Z)) \quad (5)$$

$$\text{Subtraction: } \emptyset\_(A - B)(X,Y,Z) = \max(\emptyset\_A(X,Y,Z), -\emptyset\_B(X,Y,Z)) \quad (6)$$

$$Union = \emptyset_{(A\cup B)(X,Y,Z)} = \min\,(\emptyset\_A(X,Y,Z), \emptyset\_B(X,Y,Z)) \quad (7)$$

In this work, $f_{G1}(X,Y,Z)$ and $f_{G2}(X,Y,Z)$ are two solid gyroid sheets with single scale, featuring the cell sizes of L_1 and L_2, and constant value of C_1 and C_2 respectively. The dual-scale Gyroid microlattices are modeled via Boolean intersection: $f_{GG}(X,Y,Z) = \max\,(f_{G1}(X,Y,Z), f_{G2}(X,Y,Z))$. By changing L and C at two scales, geometric properties of dual-scale structures, such as relative density and aspect ratio, can be modified.

Supplementary Note 3.

Computational insights into the NO_3RR activity origin on multi-elemental synergy of FeCoNi.

To elucidate the correlation between the synergistic effects induced by multi-elemental mixing in MEA and their electrocatalytic performance, we conducted density functional theory (DFT) calculations. We first analyzed charge density distributions for surface atoms in both pure metallic systems and the MEA to examine how multiple elements influence local electronic structure at atomic scales (Supplementary Fig. 23a). Notably, the FeCoNi MEA displayed substantial charge redistribution relative to single-element metallic structures. In particular, electron density values for Fe atoms in the MEA were reduced relative to their pure metal states, whereas Co and Ni demonstrated increased localized electron concentrations within the alloy matrix. This redistribution pattern indicates a directional electron transfer process from Fe constituents to Co/Ni elements, aligning with established research findings [8-10]. Moreover, Bader charge analysis was employed to quantify charge variations for surface atoms within the FeCoNi MEA (Supplementary Fig. 23b). While all metallic constituents displayed consistent trends in electronic structure modifications, the magnitude of charge transfer varied significantly across elements, influenced by their distinct coordination environments[11]. Fe atoms exhibited a charge gain ranging from 0.05 to 0.14 |e|, whereas Co and Ni atoms experienced a charge loss from 0.001 to 0.05 |e| and 0.04 to 0.12 |e|, respectively."

Partial projected density of states (PDOS) analysis further confirmed significant electron redistribution in the multi-component system (Supplementary Fig. 23c). Fe sites dominate near the Fermi level (EF), while Co and Ni occupy deeper energy levels. The 3d-orbitals of these elements form a near-continuous distribution via d-d hybridization, enabling efficient electron transfer in the FeCoNi MEA[12]. Element-specific PDOS and d-band center models were analyzed to explore adsorbate-alloy electronic interactions (Supplementary Fig. 23c)[13]. The d-band centers (εd) of pure Fe, Co, and Ni relative to EF were calculated as −1.04, −1.12, and −1.20 eV, respectively, indicating stronger adsorption by Fe. In the MEA, Fe (εd = −0.87 eV) exhibits d-states closer to EF, enhancing NO_3RR intermediate adsorption. Conversely, Co (εd = −1.14 eV), and Ni (εd = −1.34 eV) show downshifted d-band centers, reducing adsorption strengths. These electronic variations create a broad adsorption energy landscape in the MEA, ideal for multi-step NO_3RR catalysis. The system's ability to accommodate diverse binding energies makes it highly promising for complex catalytic applications.

Theoretical calculations are further applied to illustrate the reactivity properties of multiple active sites in MEA for NO_3RR. Thermodynamically, strong affinity for NO_3^- is a critical prerequisite for efficient NO_3^--to-NH_3 conversion[12]. The adsorption of bridge-bidentate $*NO_3$ on 9 unique coordination environments of FeCoNi catalyst was studied (Fig. 5b and Supplementary Fig. 33-36), which presents more types of active sites than the single metal slab. The nine adsorption sites of the FeCoNi-LBL slab exhibit a wide range of ΔG_{*NO3} values, spanning from -0.01 to -1.26 eV. However, adsorption energy scaling in multi-step NO_3RR restricts using $*NO_3^-$ adsorption as a full activity metric for the conversion process. Bayesian chemisorption analyses reveal linear $*NO_3$-$*N$ adsorption energy correlations in metals, establishing these as key reactivity descriptors[13]. Monometallic systems show strong $*NO_3$-$*N$ scaling (slope = 1.41, R^2 = 0.96), matching the theoretical prediction of 1.53 (Supplementary Fig. 24). MEAs, however, host heteronuclear sites

(e.g., Co-Fe, Co-Ni) that disrupt scaling laws. Simulations indicate Fe sites in MEAs stabilize $*NO_3$ via higher d-band centers, while Co/Ni sites with lower d-band positions cause repulsive interactions. This decouples $*NO_3$ and *N binding energy tuning, overcoming scaling limitations. This multi-component system, characterized by an expanded adsorption energy range, achieves an optimal balance between intermediate adsorption and desorption, thus overcoming scaling constraints to enable efficient NH_3 electrosynthesis.

Supplementary Note 4:

Discussions of DEMS and *in situ* Raman spectroscopy results

To identify the most possible NH_3 synthesis path, DEMS was carried out to detect the molecular intermediates (Fig. 4d). During four continuous LSV scan cycles, the mass-to-charge (m/z) signals corresponding to NH_3 (m/z 17), NO (m/z 30), NH_2OH (m/z 33), and NO_2 (m/z 46) were detected, with NH_3 being the dominant signal, confirming its high production rate.

However, those species were still insufficient to clarify the overall pathway. Therefore, *in situ* Raman spectroscopy was conducted to further identify reaction intermediates. As illustrated in Fig. 4e, within the potential range of −0.2 to −0.9 V, several peaks corresponding to intermediates adsorbed on the catalyst surface were observed. At open circuit potential (OCP), the initial characteristic peaks at around 998 cm^{-1} and 1049 cm^{-1} were attributed to SO_4^{2-} and NO_3^- stretching, respectively. With the increase of the applied voltage, the $*NO_3^-$ peak appeared around 1030 cm^{-1}, indicating the adsorption of NO_3^- on the surface of FeCoNi-S4650. Moreover, the peaks of NO stretching vibration emerged near 990 cm^{-1}, and symmetric bending vibrations of HNH were visible near 1315 and 1340 cm^{-1}. The symmetric bending vibrations of adsorbed $*NH_3$ at around 1591 cm^{-1} appeared as the working potential shifted negatively to −0.5 V and the intensity of this peak further increased from −0.5 V to −0.9 V, which confirmed the formation of NH_3[14]. The Raman spectra on FeCoNi-LBL catalyst indicates that the peak related to *NO, $*NH_2$ and $*NH_2$ appeared at higher overpotential (−0.6 V) due to its poor ability for deep $*NO_3^-$ hydrodeoxidation properties. Moreover, the band's intensities of all the detected intermediates formed on FeCoNi-LBL were very weak, meaning that a low amount of intermediates (*NO and $*NH_2$) and product ($*NH_3$) were generated (Supplementary Fig. 32).

Supplementary Note 5:

Mechanical behavior of FeCoNi dual-scale gyroid

Architecture design advantages

The strain-stress curve depicted in Fig. 6a for FeCoNi dual-scale gyroid exhibits the typical three stages of homogeneous deformation without a pronounced stress drop, encompassing the initial linear-elastic stage, followed by the plateau stage, and culminating in the densification stage. Finite element method revealed that dual-scale gyroid displays similarly smooth stress distribution but with a much higher mean Gaussian curvature compared to single-scale gyroid (Supplementary Fig. 40). From the *in situ* SEM compression images in Fig. 6b, our dual-scale gyroid are not only drastically stronger (over 3 times) than the commercial Ni foam, but also exhibit a more homogeneous deformation, displaying a near layer-by-layer collapse with little to no thin wall fracture over 50% strain (near its densification strain of 57%). In contrast, commercial Ni foam displayed low mechanical strength and strain localization with a shear band oriented at 45° angle concerning to the loading direction, followed by localized plastic bulking until densification. The comparison of stress-strain curves between Ni dual-scale gyroid and Ni foam proves that our architectural design played an important role in enhanced mechanical strength (Supplementary Fig. 41).

Materials design advantages

The stress-strain curve in Supplementary Fig. 41, the FeCoNi dual-scale gyroid exhibited enhanced compressive strength and specific strength (over 2 times) compared to pure Ni dual-scale gyroid. We further observed the fracture microstructure using SEM image. The fracture surface morphologies of the samples subjected to FeCoNi metamaterials displayed numerous dimples, indicating a ductile fracture mode. Conversely, quasi-cleavage fracture morphology was observed at Ni metamaterials, demonstrating the brittle fracture mode (Supplementary Fig. 41). This demonstrates that our multicomponent composition design effectively enhances the strength and ductility of the dual-scale gyroid metamaterials. The energy absorption and specific energy absorption, calculated from the area under the corresponding stress-strain curves, revealed an over 50% increase in the FeCoNi dual-scale gyroid compared to the pure Ni dual-scale gyroid. Previous studies found that, enhanced mechanical properties in FeCoNi alloy may be attributed to the severe lattice distortion [15] and the sluggish diffusion effect in multicomponent alloys [16].

Supplementary Method 1:

Geometric phase analysis

Geometric phase analysis represents a digital signal processing technique employed to quantify displacements and strain fields with atomic scale resolution by extracting the real-space and Fourier-space information of an HRTEM or HAADF-STEM image. According to the previous geometric phase analysis methodology[17], two diffraction spots with nonlinear correlation within the diffraction patterns from the HRTEM images were employed to delineate the two-dimensional HRTEM images in real space, facilitating the measurement of lattice distortion within the high-resolution phase. We used the strain++ software to establish the strain mapping of HRTEM images. The diffraction spots we used were (200) and $(02\bar{2})$ along the face-centred cubic zone [110] axis.

Supplementary Method 2:

Dislocation density calculation

The dislocation density was calculated using the Williamson-Hall method[18]. Internal strain within the samples was assessed through X-ray diffraction (XRD) pattern analysis. The XRD experiments were conducted with a Rigaku SmartLab X-ray diffractometer, noted for its exceptional resolution and a 2θ angle range of 40° to 80°. The scans were performed at a speed of 0.1°/min with a step size of 0.02°. Instrument broadening was calibrated using a strain-free silicon powder as the reference.

$$\delta cos\theta = \frac{\lambda}{D} + 2\varepsilon\, sin\theta \tag{8}$$

where δ is the physical broadening of full width at half maximum (FWHM) of the diffraction peak, θ is the diffraction angle, λ is the wavelength of radiation, D is the grain size, and ε is the internal strain. The linear fit may be used to get the ε and D. Grain size has a negligible effect on peak broadening. (the wavelength $\lambda_{K\alpha 1}$=0.154nm and D=~2.8μm). Hence, the term of λ/D can be close to zero. For linear fitting, three planes of (111), (200), and (220) were used. The dislocation density ρ was calculated as below:

$$\rho = k\frac{\varepsilon^2}{b^2} \tag{9}$$

where k=16.1 for FeCoNi[19], and the Burgers vector b is 0.2525nm.[20]

Supplementary Method 3:

Electrochemical in situ Raman spectroscopy

In-situ Raman measurements were performed using a custom-made Teflon reactor on a WITec alpha300 R Raman System with a 532 nm laser as the excitation source. The shell-lattice metamaterial catalysts, Pt wire and Ag/AgCl served as working electrode, counter electrode and reference electrode, respectively. In situ Raman spectra were recorded in electrolytes with NO_3^- under control by an electrochemical workstation. In detail, the potentials were set from open circuit potential (OCP) to −0.7 V vs. RHE. Each potential was kept for 300 s and then collected the Raman spectra with a collection time of 20 s.

Electrochemical online DEMS tests

To accurately identify the intermediates and products, DEMS measurements were carried out on a customized testing system, which consists of an electrochemical reactor and a mass spectrometer. The electrolyte and three-electrode setup were similar to the electrochemical tests. Typically, LSV scanning was carried out at a speed of 5 mV s^{-1} and a voltage range of -0.1 ~ -1.3 V and kept vacuum system of the mass spectrometer closing. After the baseline remains constant, open vacuum system of mass spectrometer to capture the signal of the generated gas molecules during the electrocatalytic process. The mass spectrometer's sampling probe was positioned in close proximity to the working electrode in order to promptly catch gas molecules.

Quantitation of nitrate using UV-vis

The concentration of NO_3^- was quantitatively determined by UV–vis spectrophotometry according to the standard method. Firstly, a certain amount of electrolyte samples was collected and diluted to a final volume of 10 mL to the detection range. Then, 1 ml of 1 M HCl with 0.1 ml of 0.8wt% sulfamic acid solution was added to the diluted sample and shaken to obtain a uniform solution. The absorption intensities at wavelengths of 220 and 275 nm were then recorded by an ultraviolet-visible (UV–vis) spectrophotometer. The final absorbance value A is calculated using the equation: A $=A_{220nm} - 2 \times A_{275nm}$. The concentration–absorbance curve was constructed through calibration with a series of standard potassium nitrate (KNO_3) solution. The NO_3^- concentration was then tested according to the calculated absorbance value and the standard calibration curve.

Quantitation of nitrite using UV-vis

The concentration of NO_2^- was quantitatively determined by UV-vis spectrophotometry using the Griess method. A specific color reagent of this method was prepared by mixing N-(1-Naphthyl) ethylenediamine dihydrochloride (1 g), p-aminobenzene sulfonamide (20 g), deionized water solution (250 mL), and phosphoric acid (50 mL). Then, 0.1 ml color reagent was added into 4.9 mL diluted electrolyte and mixed uniformity. After sitting for 20 min, the absorption intensity of NO_2^- at a wavelength of 540 nm was recorded. The concentration-absorbance curve was calibrated using a series of standard sodium nitrite (KNO_2) solutions.

Quantitation of ammonium using UV-vis

The concentration of $NH4^+$ was determined by the indophenol blue approach. First, 0.5 ml of electrolyte was taken out from the electrolytic cell and diluted to 3 ml to the detection range. Then, 1 mL of NaOH (2.0 M) containing sodium citrates ($C_6H_5Na_3O_7 \cdot 2H_2O$, 10.0 wt%) and salicylic acid

($C_7H_6O_3$, 10.0 wt%) was injected. Thereafter, 1 mL of NaClO (0.05 M) solution and 0.2 mL of sodium nitroferricyanide ($Na_2[Fe (NO)(CN)_5] \cdot 2H_2O$, 1.0 wt%) solution were added and mixed evenly. The absorbance value at a wavelength of 658 nm was recorded after maintaining for 2 h. The absorbance-concentration curve was calibrated using a series of standard ammonium chloride ($NH_4(SO_4)_2$) solutions which dried at 110 °C for 2 h. The concentration-absorbance calibration curve was calibrated using a series of standard concentration ammonium sulfate $NH_4(SO_4)_2$ solutions.

Quantitation of ammonia using ^{1}H NMR

The isotope-labeling nitrate reduction experiments were analyzed by ^{1}H NMR. First, 0.10 M $Na^{15}NO_3$ /0.50 M Na_2SO_4 was used as the electrolyte to clarify the source of NH_3. The obtained electrolyte (25 ml) after electroreduction was adjusted to be weak acid with H_2SO_4 and then adding maleic acid ($C_4H_4O_4$, 0.01 g) as an internal standard.

After that, 0.5 ml of the above mixtures was mixed with 50 μl D2O for the ^{1}H NMR test. A series of $^{15}NH_4^+$-^{15}N solution (25 ml) with known concentrations were prepared to obtain the calibration curve. Similarly, $Na^{14}NO_3/Na^{15}NO_3$ equals to 1:1 was also used as the feeding N-source in electrocatalytic reaction, then the ratio of products $^{14}NH_4^+$-^{14}N/ $^{15}NH_4^+$-^{15}N was tested by the same method as above-mentioned ^{1}H NMR detection to further clarify the source of ammonia.

Supplementary Method 4:

Calculation of the Faradaic efficiency, yield rate, and energy efficiency

Faradaic efficiency of NH_3 was calculated by:

$$FE_{NH3}(\%) = \frac{8F \times c_{NH_3} \times V}{17 \times Q} \quad (10)$$

Faradaic efficiency of NO_2 was calculated by:

$$FE_{NO2}(\%) = \frac{2F \times c_{NO_2} \times V}{46 \times Q} \quad (11)$$

NH_3 yield rate was calculated by:

$$NH_3 \text{ yield rate } (mg\ h^{-1}\ cm^{-2}) = \frac{c_{NH_3} \times V}{t \times s} \quad (12)$$

where F is Faraday constant (96485 C mol^{-1}), c_{NH3} and c_{NO2} (mg L^{-1}) are the NH_3 concentration and NO_2 concentration after electrolysis, V (mL) is the volume of the cathodic electrolyte, Q (C) is the total charge passing the electrode, t is the reduction time, s is the geometric area of catalyst (cm^2).

The half-cell energy efficiency was defined as the ratio of fuel energy to applied electrical power, which was calculated by:

$$EE_{NH3} = \frac{(E^{\theta}_{OER} - E^{\theta}_{NH3}) \times FE_{NH3}}{E_{OER} - E_{NH3}} \quad (13)$$

E^{θ}_{OER} is the equilibrium potential of OER (1.23 V versus RHE). E^{θ}_{NH3} represents the equilibrium potential of nitrate electroreduction to ammonia, which is reported as E^{θ}_{NH3} = 0.69 V vs. RHE under alkaline conditions (pH 14) [21]. The Pourbaix diagram for ammonia provides the equilibrium potentials at different pH values [22-24]. FE_{NH3} is the Faradaic efficiency for NH_3. E_{OER} is 1.23 V versus RHE (assuming the overpotential of the water oxidation is zero), and E_{NH3} is the applied potentials in NO_3^--to-NH_3 electroreduction [25].

Supplementary Tables:

Table S1. Linear shrinkage rate during the fabrication process

**Length at each hierarchy**	**As-printed hydrogel metamaterial**	**Final FeCoNi metamaterial**	**Shrinkage rate (%)**
Whole sample (mm)	15.1	4.2	72.2
First order pore (mm)	3.1	0.9	71.0
Second order pore (μm)	414.2	117.6	71.6
Minimum wall thickness (μm)	76.5	10.2	73.6
Average	-	-	72.1

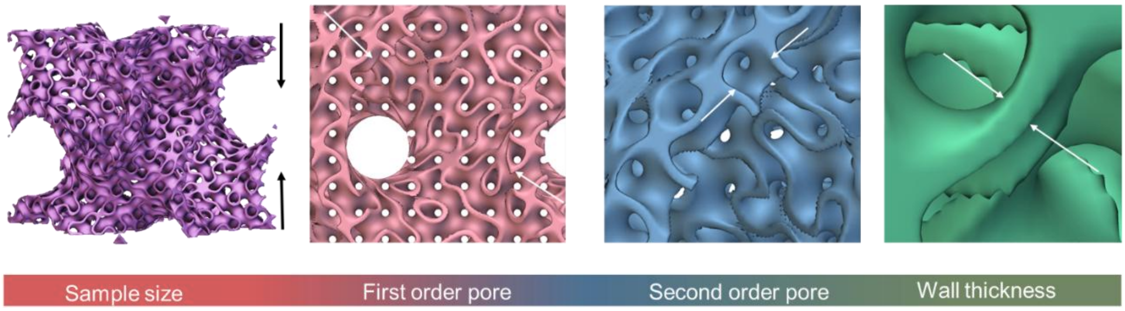

Table S2. Dislocation density comparison using different measurement methods

Estimation method	Material	Catalytic reaction	Dislocation density (m^{-2})	References
**Williamson-Hall or EBSD (macro or mesoscale)**	FeCoNi	Electrochemical nitrate reduction reaction	2.23×10^{14} (WH) 1.35×10^{14} (EBSD)	This work
	$BaTiO_3$	Water splitting	1.5×10^{14}	26
	Pure Cu	CO_2 reduction reaction	2.23×10^{14}	27
	NiCrFeMo	CO_2 methanation	1.32×10^{14}	28
**GPA analysis of HRTEM (nanoscale)**	FeCoNi	Electrochemical nitrate reduction reaction	2.2×10^{17}	This work
	Mo_2C	Hydrogen evolution reaction	1.0×10^{17}	29
	V_2O_5	Hydrazine oxidation	1.5×10^{15}	30
	PtNi/NF	Hydrogen evolution reaction	9.9×10^{17}	31

Table S3. Comparison of NO_3^--N concentration, NH_3 Faradaic efficiency, NO_3^- to NH_3 Selectivity and NH_3 yield rate for recently reported state-of-the-art NO3RR electrocatalysts.

**Materials**	**FE(NH_3)**	**NH_3 yield rate mg h^{-1} cm^{-2}**	**long-time （h）**	**References**
Metamaterial catalyst (FeCoNi-S4650)	95.4% at -0.7 V vs RHE	20.58	504	This work
Ru_1Cu_{10}/rGO	98% at -0.02 V vs RHE	6.46	12	32
Cu/Co0.85Se	93.5% at -0.6V vs RHE	2.36	600	33
CuCl@TiO2	44.7at -1.0V vs RHE	1.82	42	34
Co+Bi@Cu NW	99.51%at -0.6 V vs RHE	1.85	15	35
CuCoSP	*93.3 % ± 2.1 at -0.175V vs. RHE*	19.89	10	36
FePC/TiO_2	90.6% at -0.45V vs. RHE	17.39	24	37
Fe-PPy SACs	100% at -0.6 V vs. RHE	2.02	10	38
Co/CoO NSAs	93.8% at -1.3 V vs. SCE	3.30	10	39
Fe single atom catalyst (SAC)	75%at −0.66 V vs. RHE	1.24	35	40
CuPd aerogels	90.02% at -0.46 V vs. RHE	0.78	10	41
$Cu_{50}Ni_{50}$ alloy	93 ± 2% at - 0.2 V vs. RHE	-	12	42
Cu/Cu_2O nanowire arrays	95.8% at -0.85 V vs. RHE	4.08	12	43
PdCu/Cu_2O	94.3% at -0.8 V vs. RHE	3.23	12	44
PTCDA/O-Cu	85.9 ± 3% at - 0.4 V vs. RHE	7.34	15	45
Mo/H-CuW	94.60% at -0.7 V vs. RHE	1.46	15	46
Cu_2O/NiO	95.6% at -0.2 V vs. RHE	3.57	20	47

Ru-CuNW	99.8% at 0.04 V vs. RHE	76.5	100	[48]
Pd	35% at -0.2 V vs. RHE	0.34	4	[49]
Ru nanoclusters	100% at -0.8 V vs. RHE	19.89	100	[50]
$FeNi_{500}/FF$	65.2% at -1.1 V vs. RHE	0.26	42	[51]

Supplementary Figures:

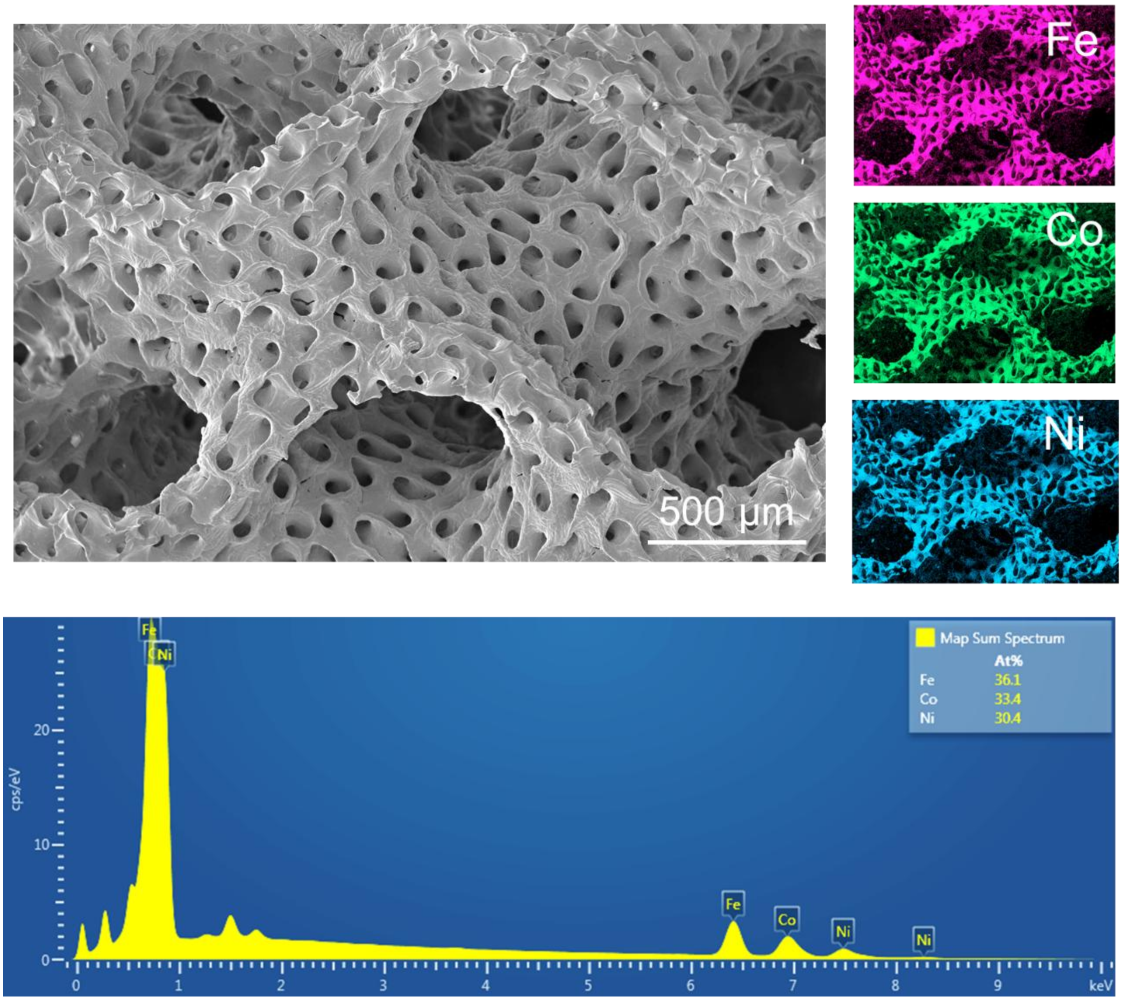

Figure S1. SEM-EDS mapping result of dual-scale gyroid showing the element distribution and corresponding atomic ratio observed from the top view.

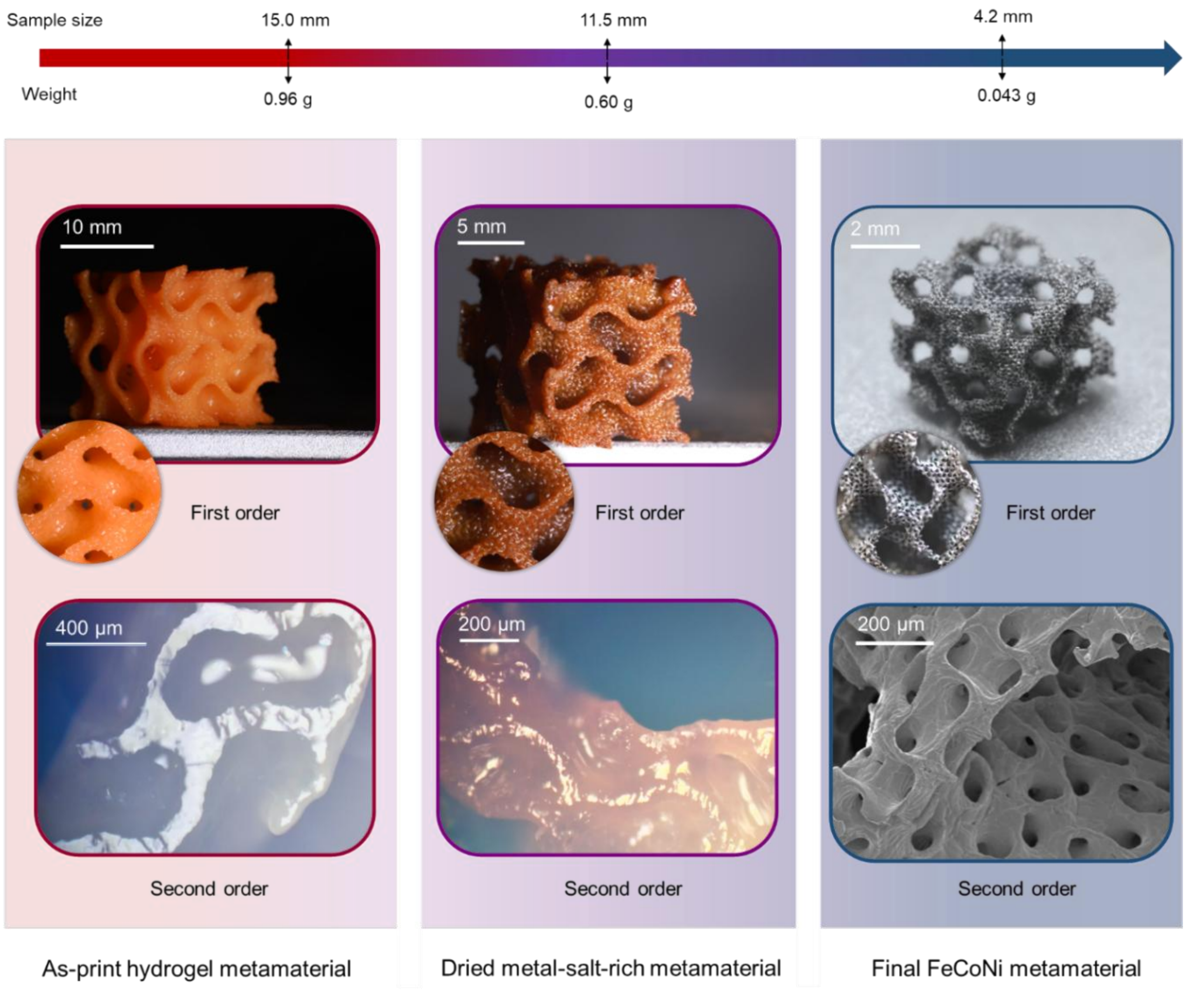

Figure S2. Geometry morphology and feature size evolution during the fabrication process

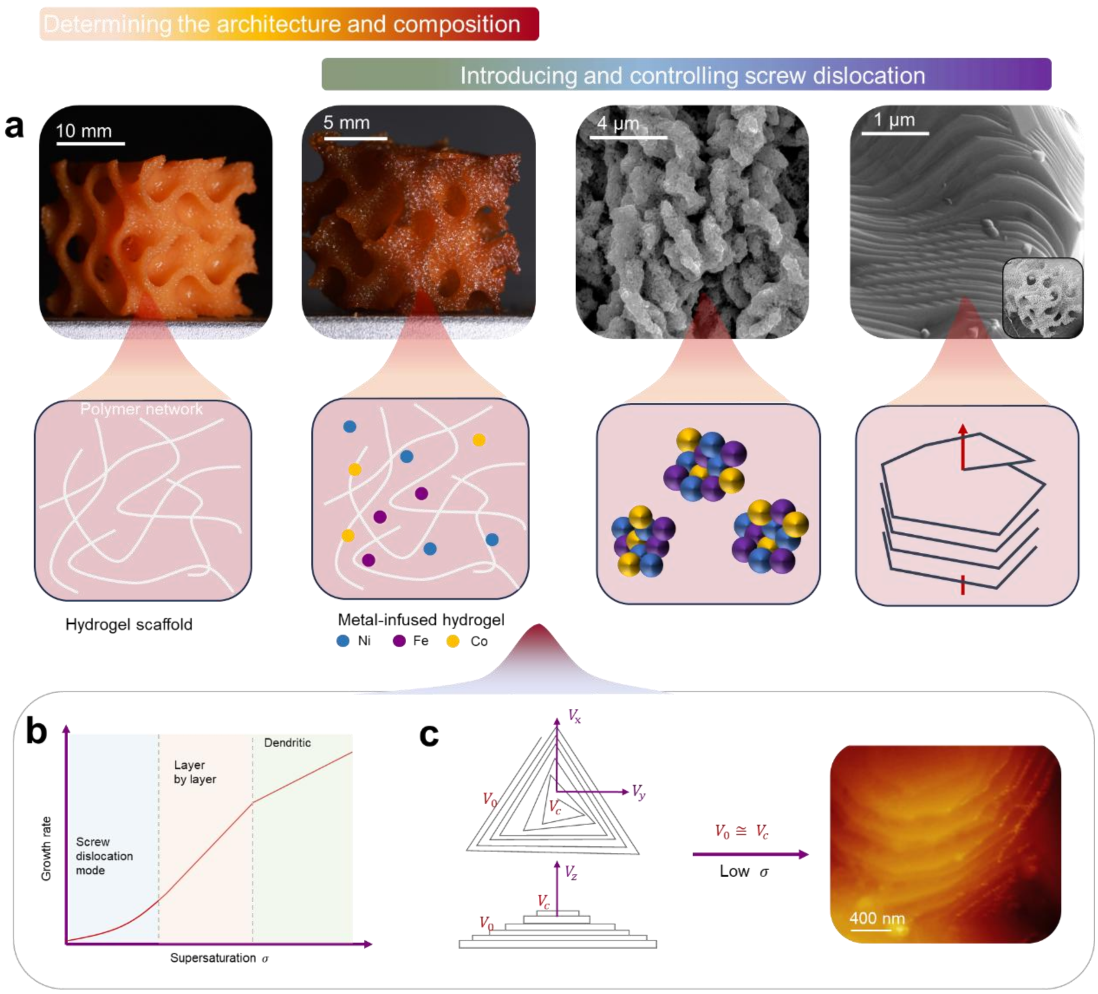

Figure S3. Mechanisms and contributions of each step in the fabrication process toward achieving interface-free metamaterial catalysts.

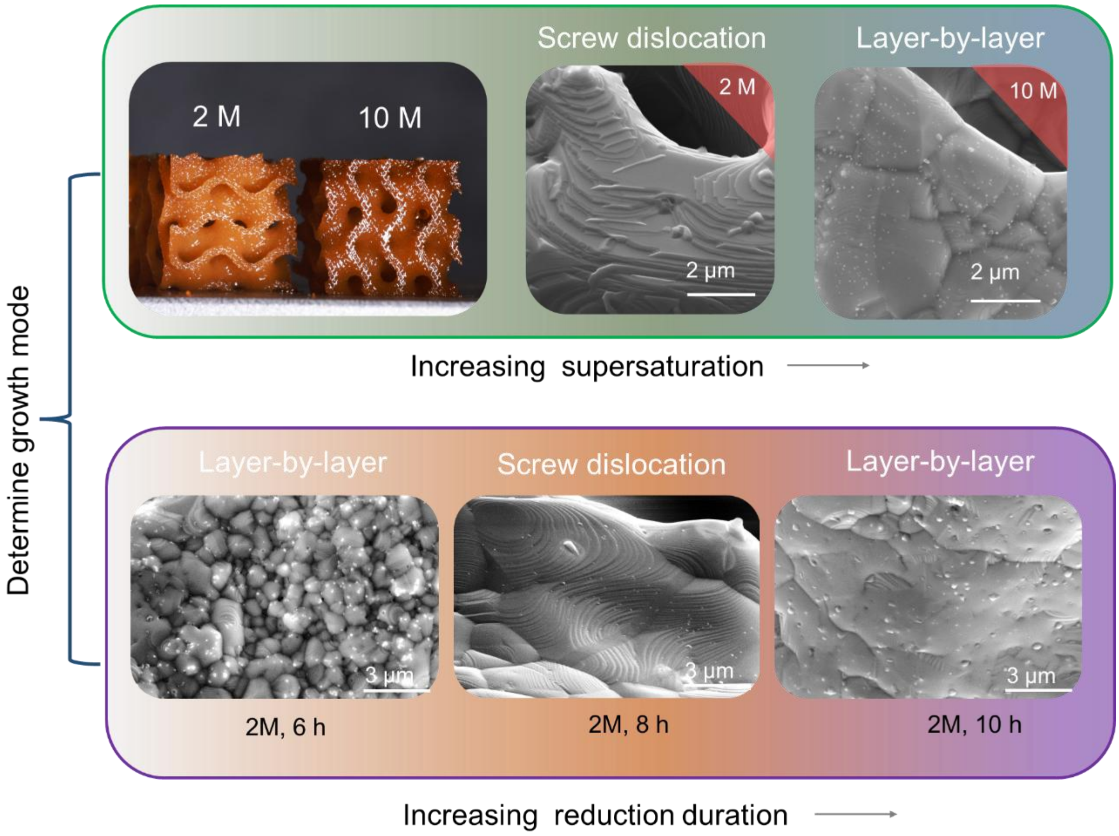

Figure S4. Synthesis parameters optimization of metal salt precursor concentration and reduction duration.

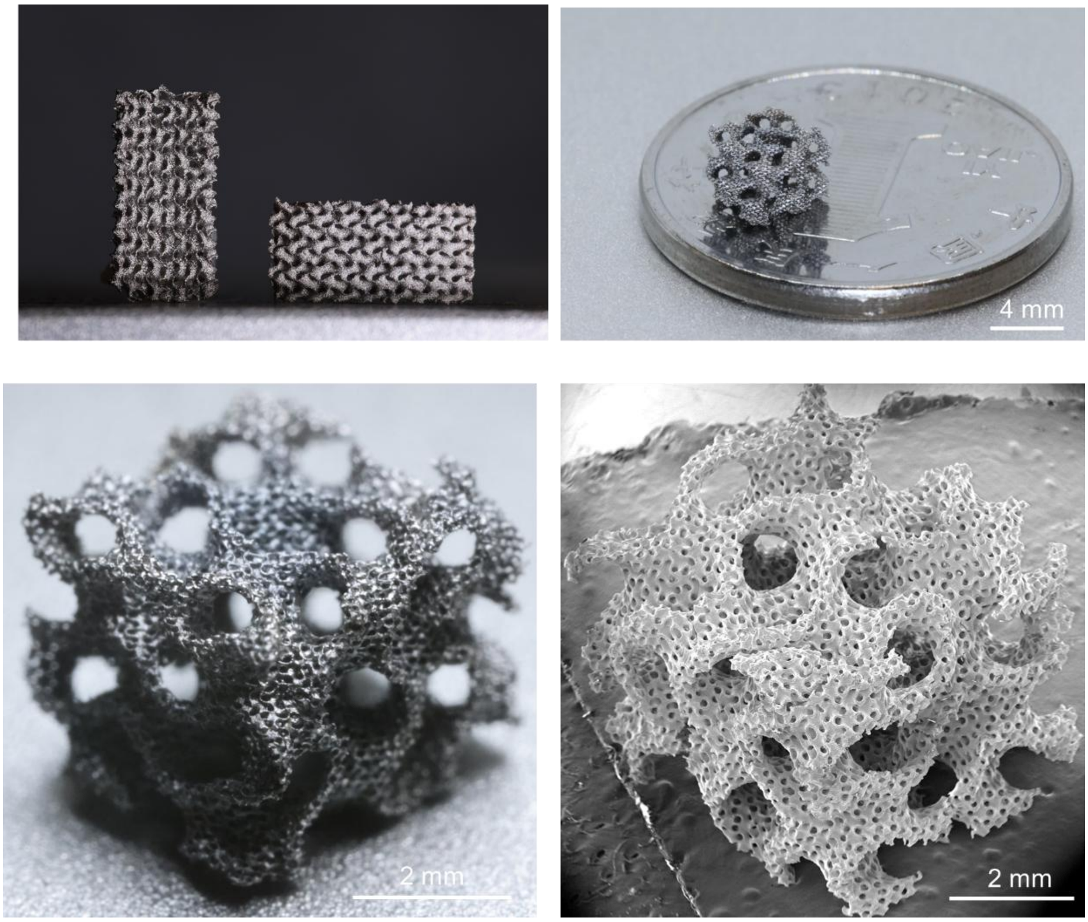

Figure S5. Camera photo and corresponding SEM image of metamaterial catalysts.

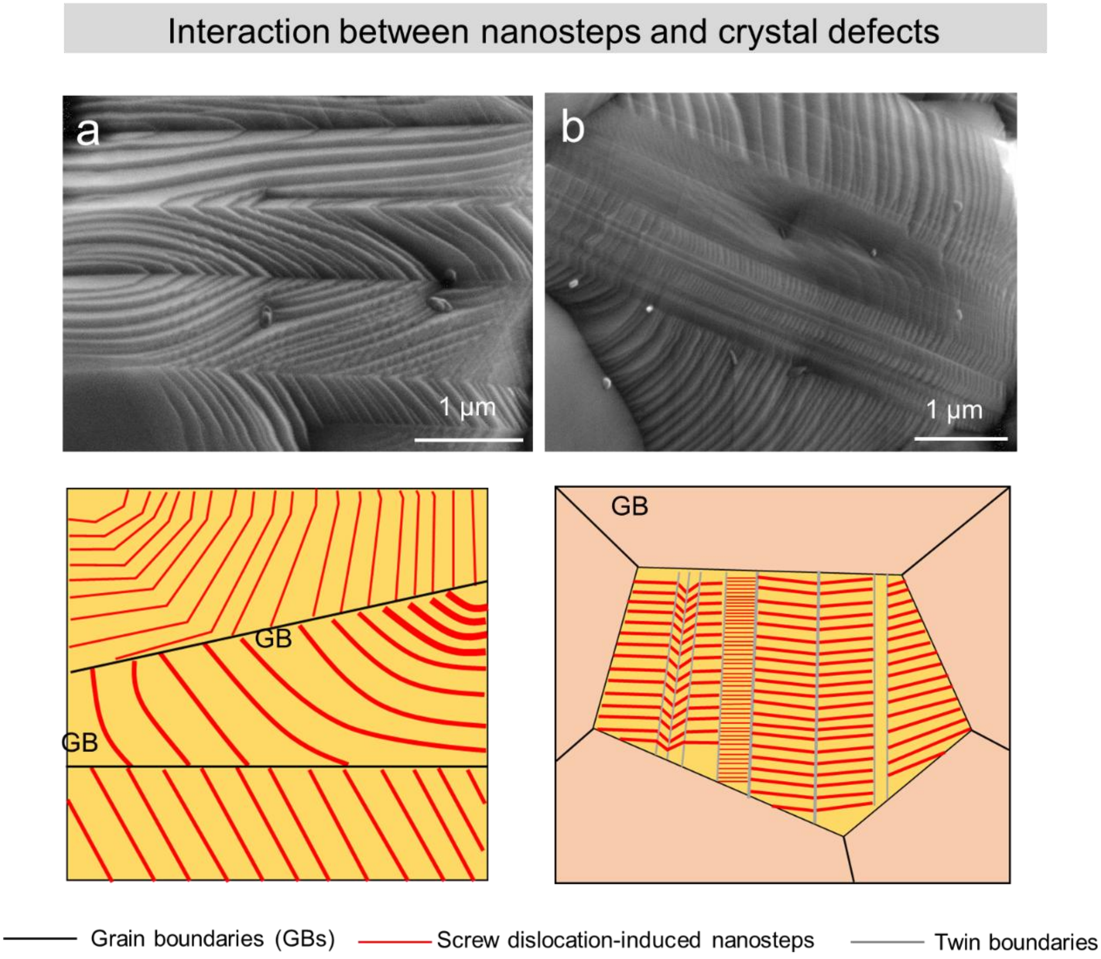

Figure S6. The interaction between nanosteps and typical crystal defect, twin boundary (a) and grain boundary (b).

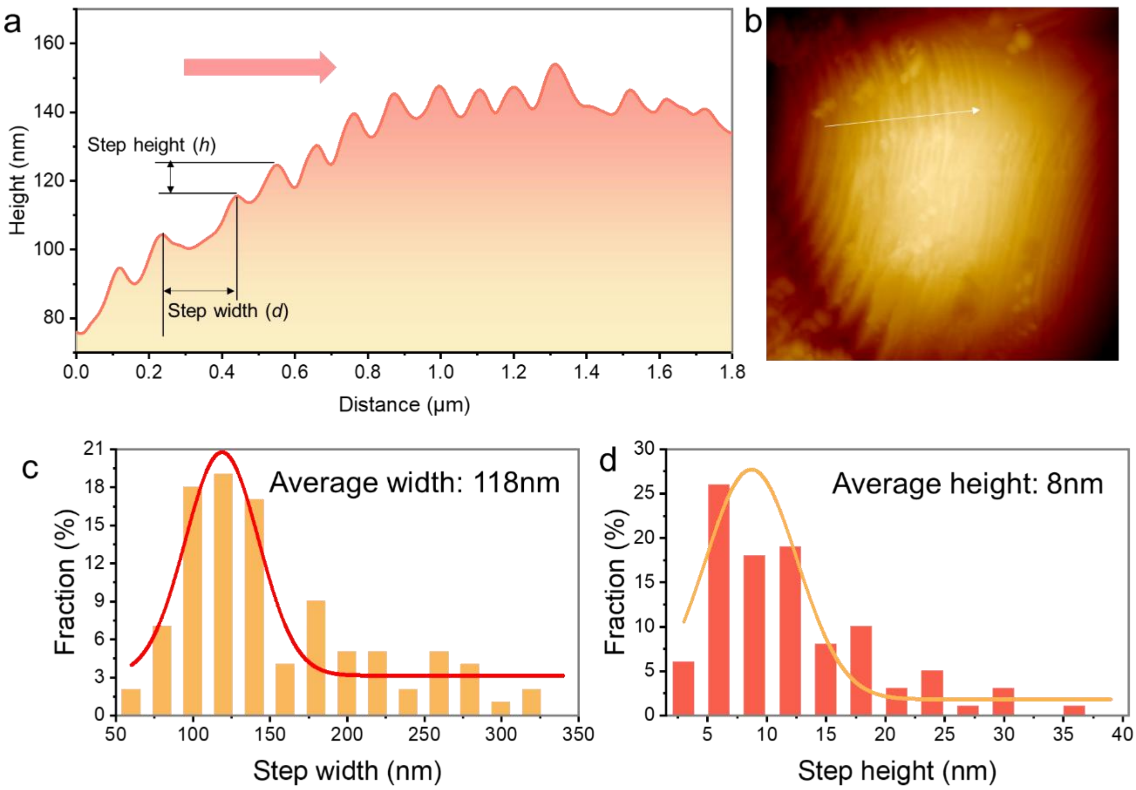

Figure S7. Atomic force microscopy (AFM) characterizations of the surface plate-like nanostructure. (a) AFM height profile of the nanoplates along the white arrow line in (b). (c) The histogram of the measured nanoplates platform width with a Gaussian fitting to the data. The Gaussian peak is centered at 118 nm. (d) The histogram of the measured nanoplates thickness with a Gaussian fitting to the data. The Gaussian peak is centered at 8 nm.

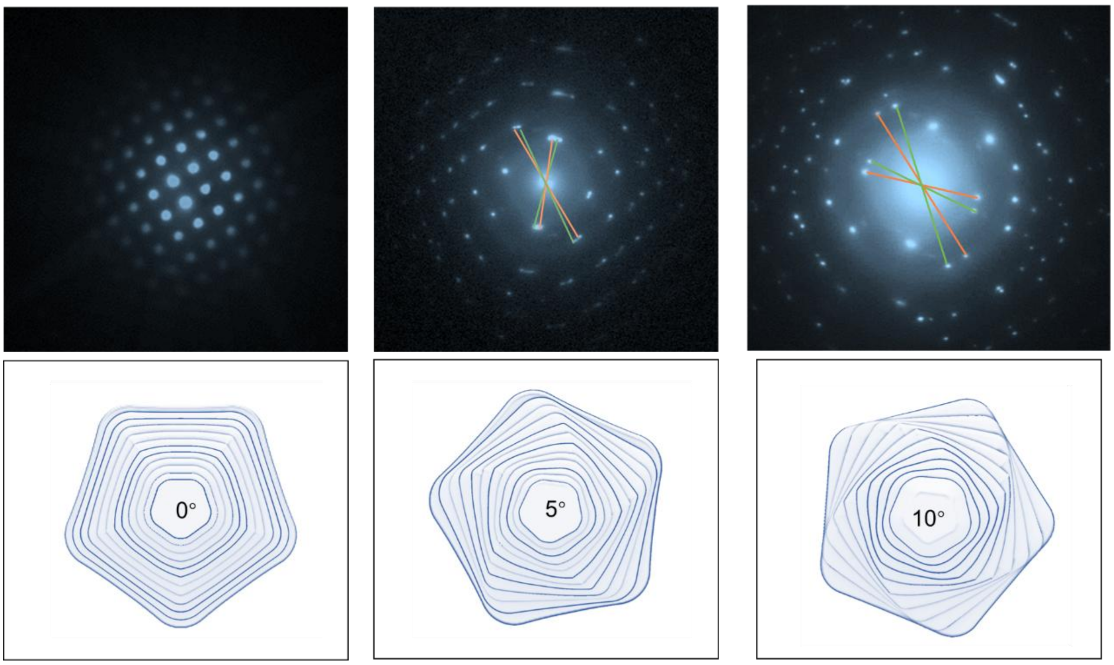

Figure S8. TEM diffraction pattern reveals the different twist angles among the nanoplates within the same grain. The schematic diagrams showing different twist angles observed from the top view.

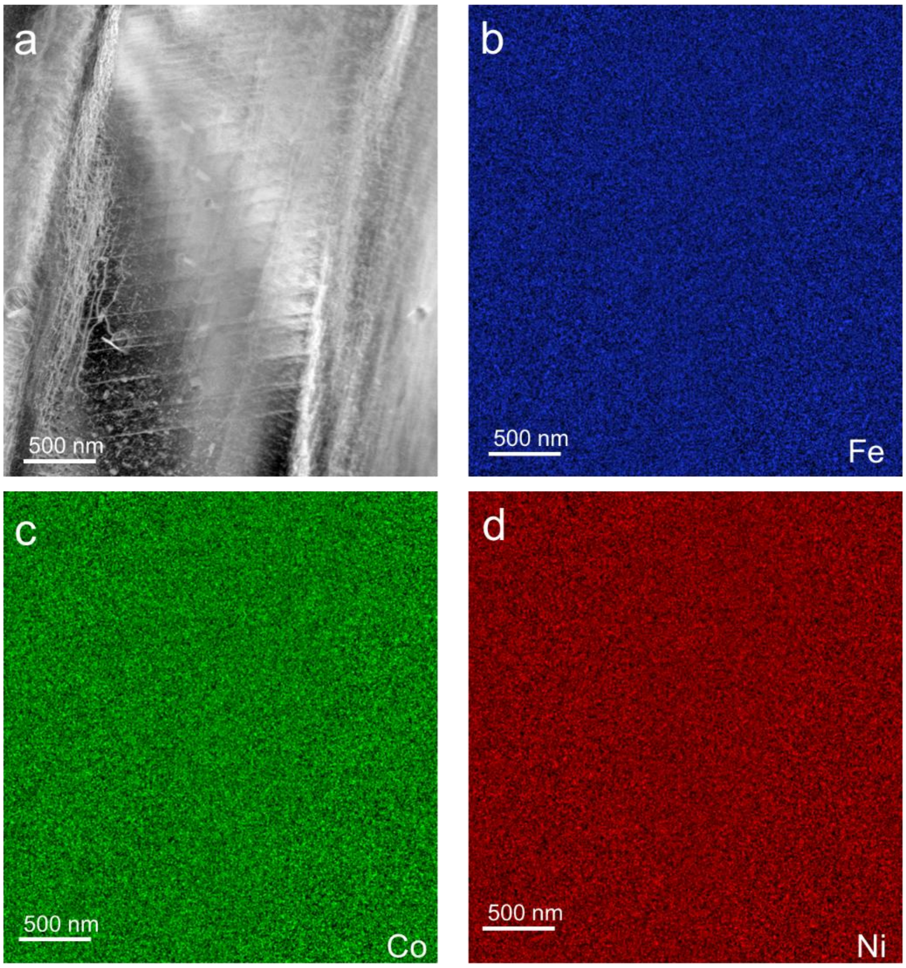

Figure S9. STEM-EDS mapping showing the homogeneous elements distribution among the nanosteps.

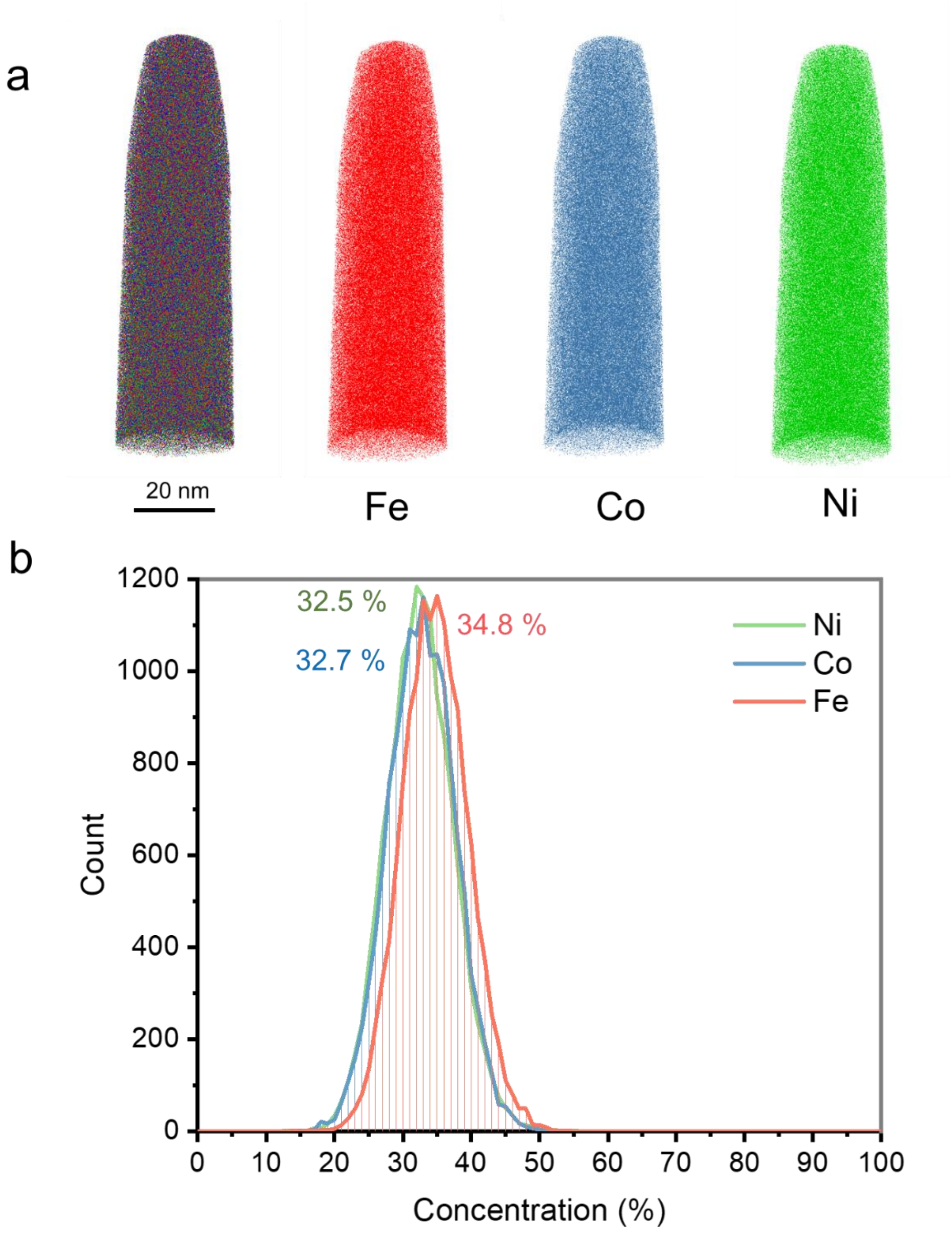

Figure S10. (a) 3D APT map showing the homogeneous elements distribution without segregation at atomic scale. (b) The concentration count distribution analysis from the APT reconstruction reveals the nearly equal atomic ratio of Fe, Co, Ni elements.

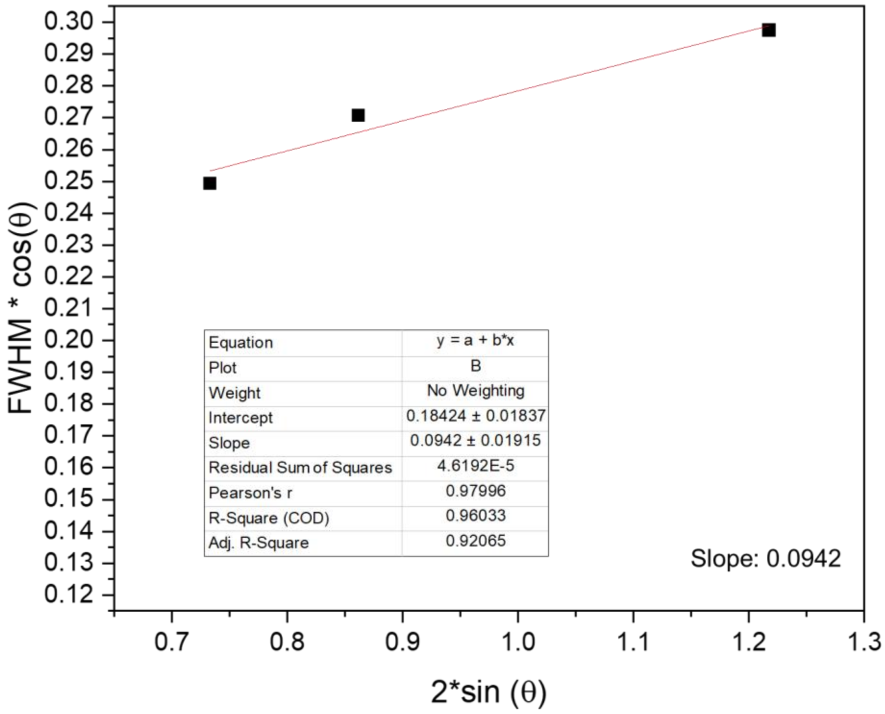

Figure S11. Williamson-Hall plot for the XRD diffraction pattern of dual-scale shell-lattice metamaterials.

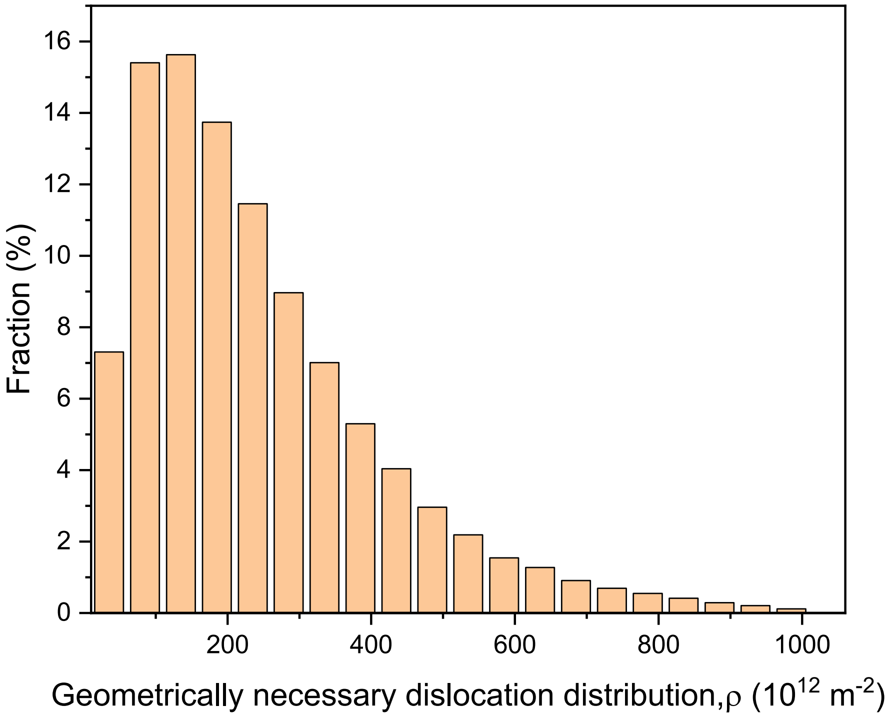

Figure S12. Geometrically necessary dislocation (GND) distribution measured by Electron backscattered diffraction (EBSD).

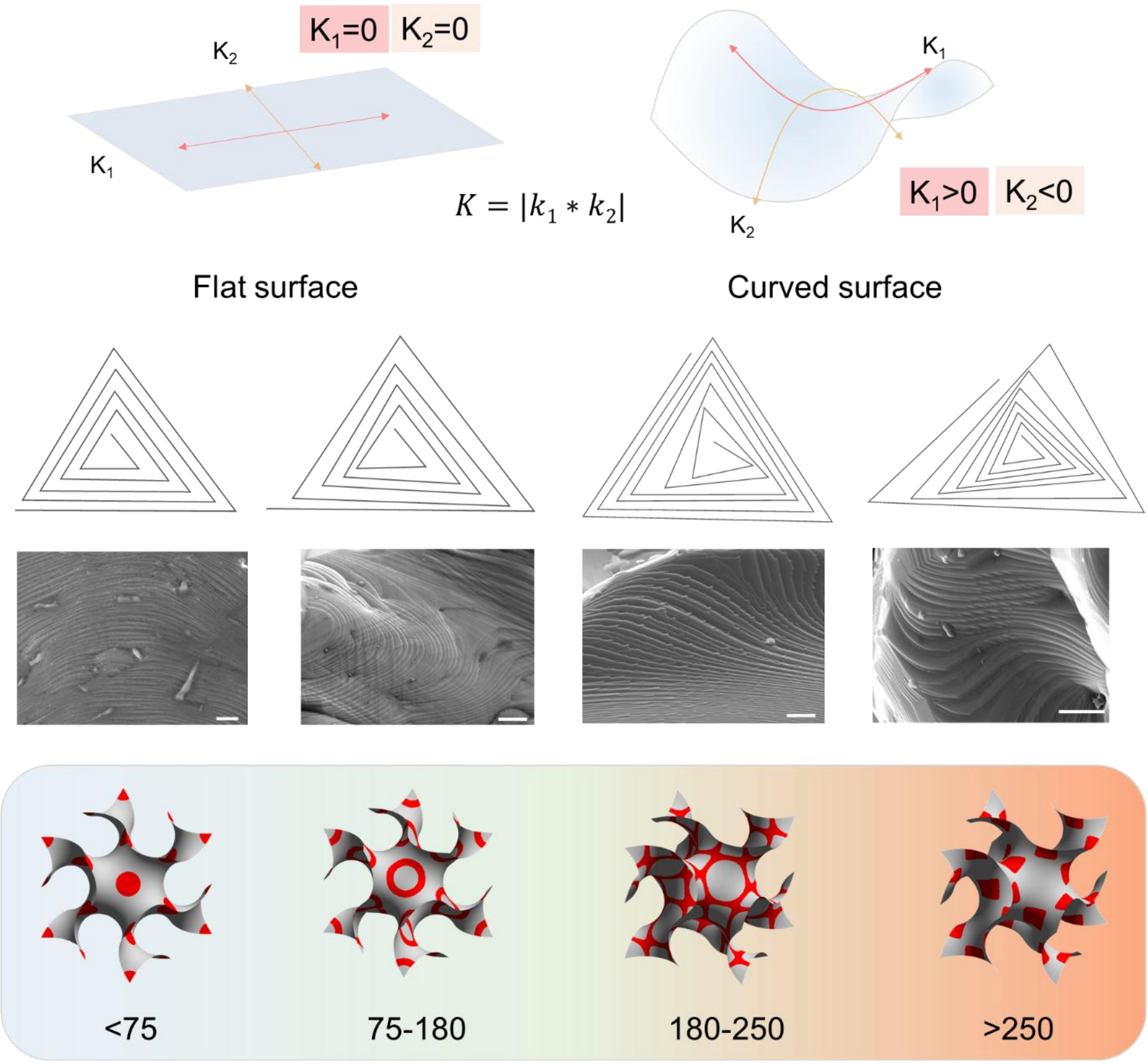

Figure S13. Influence of Gaussian curvature on the translational symmetry of nanosteps on the surface of shell-lattice metamaterials, scale bar: 1 µm.

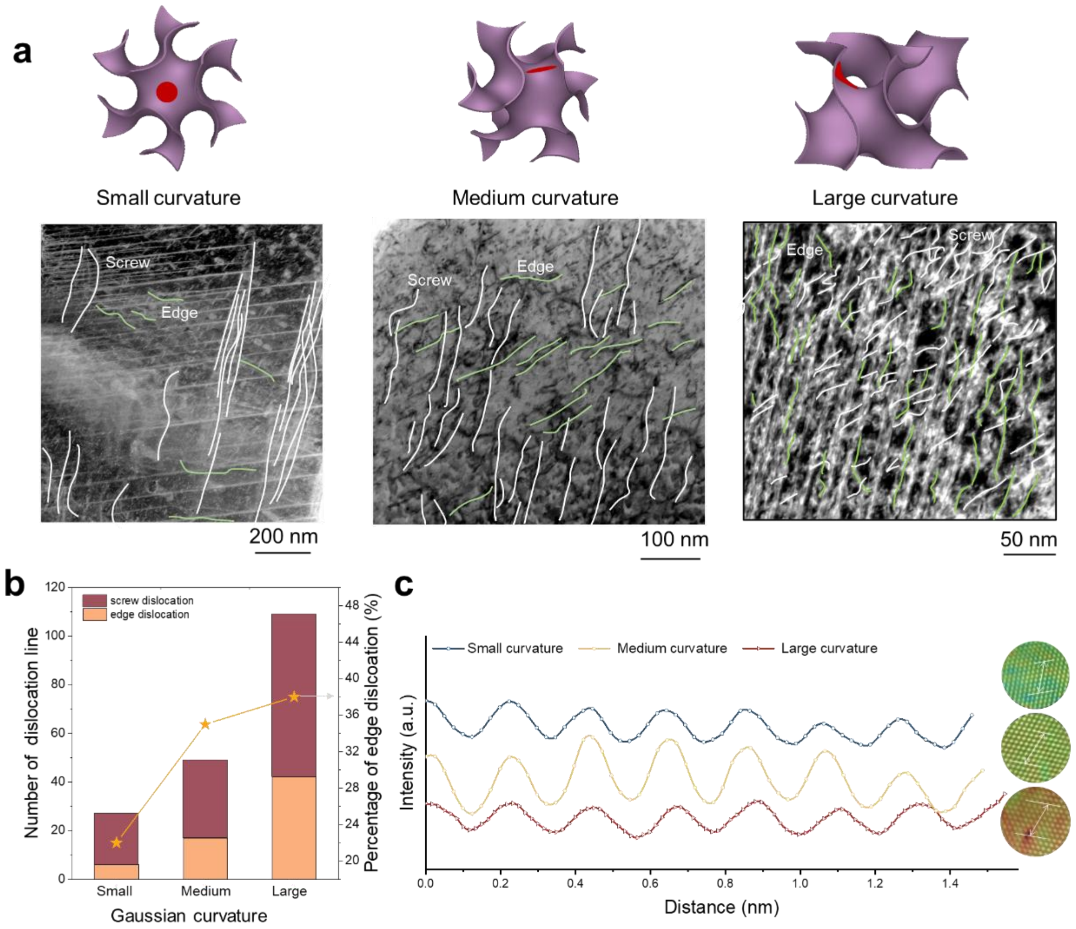

Figure S14. The influence of substrate curvature on dislocation density (a), dislocation types (b), and lattice strain (c).

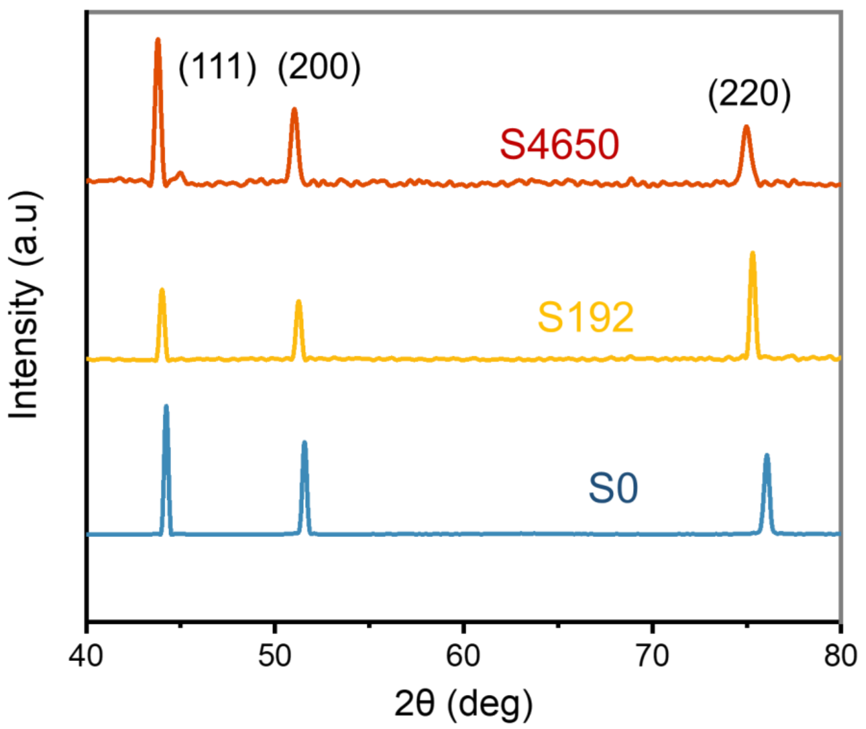

Figure S15. XRD profiles comparison of metamaterials with different average mean absolute Gaussian curvatures, FeCoNi-S0, FeCoNi-S192, and FeCoNi-S4650 samples.

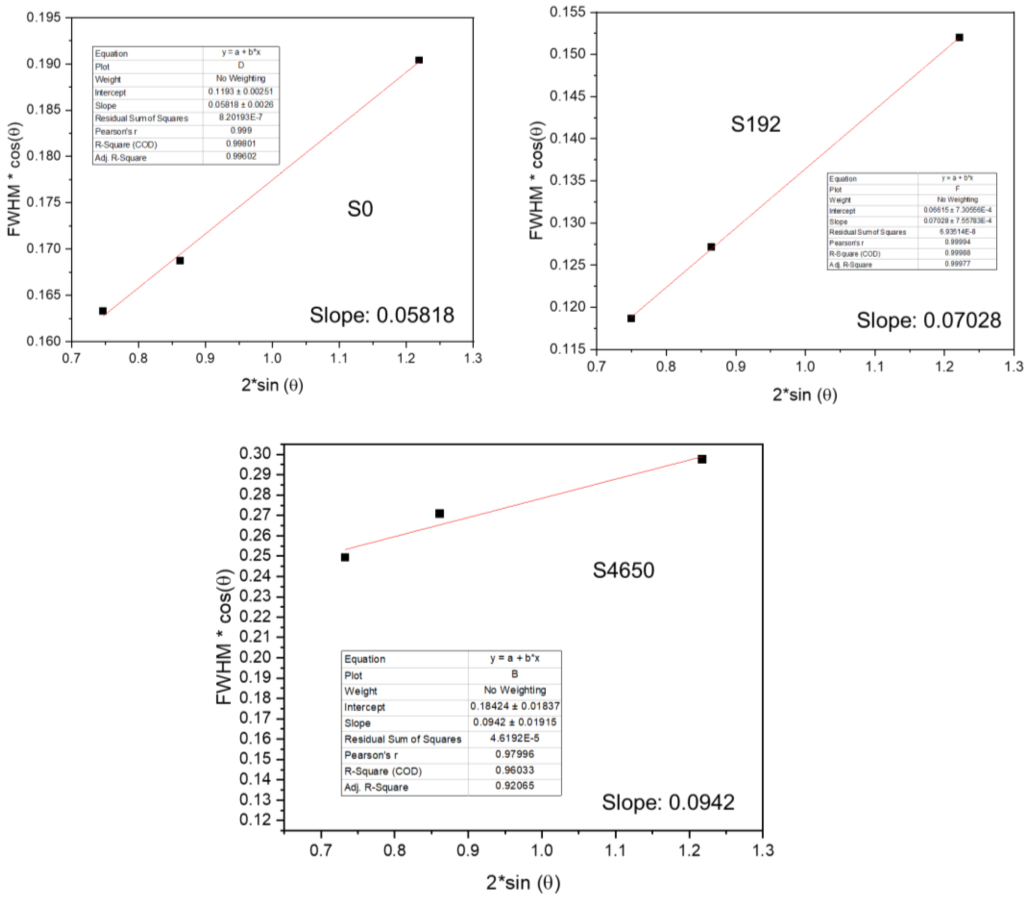

Figure S16. Williamson-Hall plot for the XRD diffraction pattern of FeCoNi-S0, FeCoNi-S192, and FeCoNi-S4650 samples.

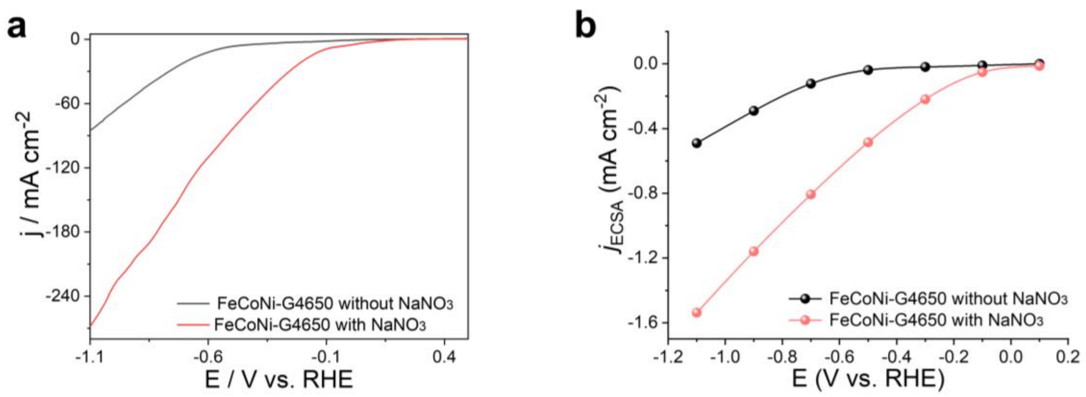

Figure S17 (a) LSV curves and (b) ECSA normalized partial current densities in LSV curves of FeCoNi-S4650 in 0.5 M Na_2SO_4 electrolyte with and without 0.1 M $NaNO_3$ upon a scan rate of 5 mV s^{-1}.

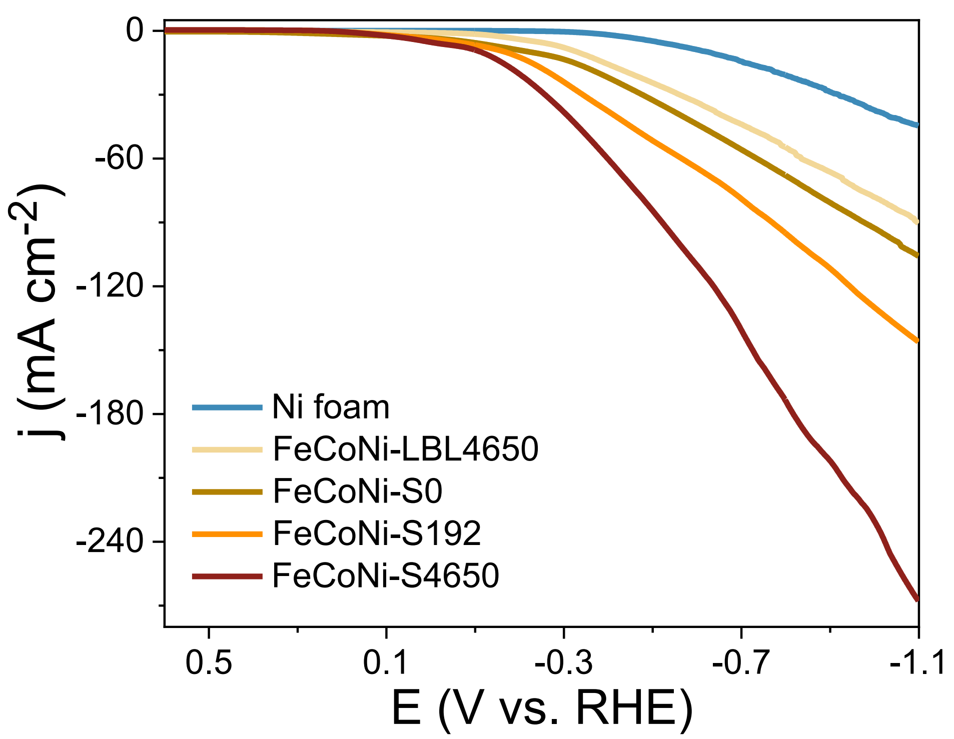

Figure S18. LSV curves of Ni foam, FeCoNi-LBL4650, FeCoNi-S0, FeCoNi-S192, and FeCoNi-S4650 in 0.5 M Na_2SO_4 electrolyte with 0.1 M $NaNO_3$ upon a scan rate of 5 mV s^{-1}.

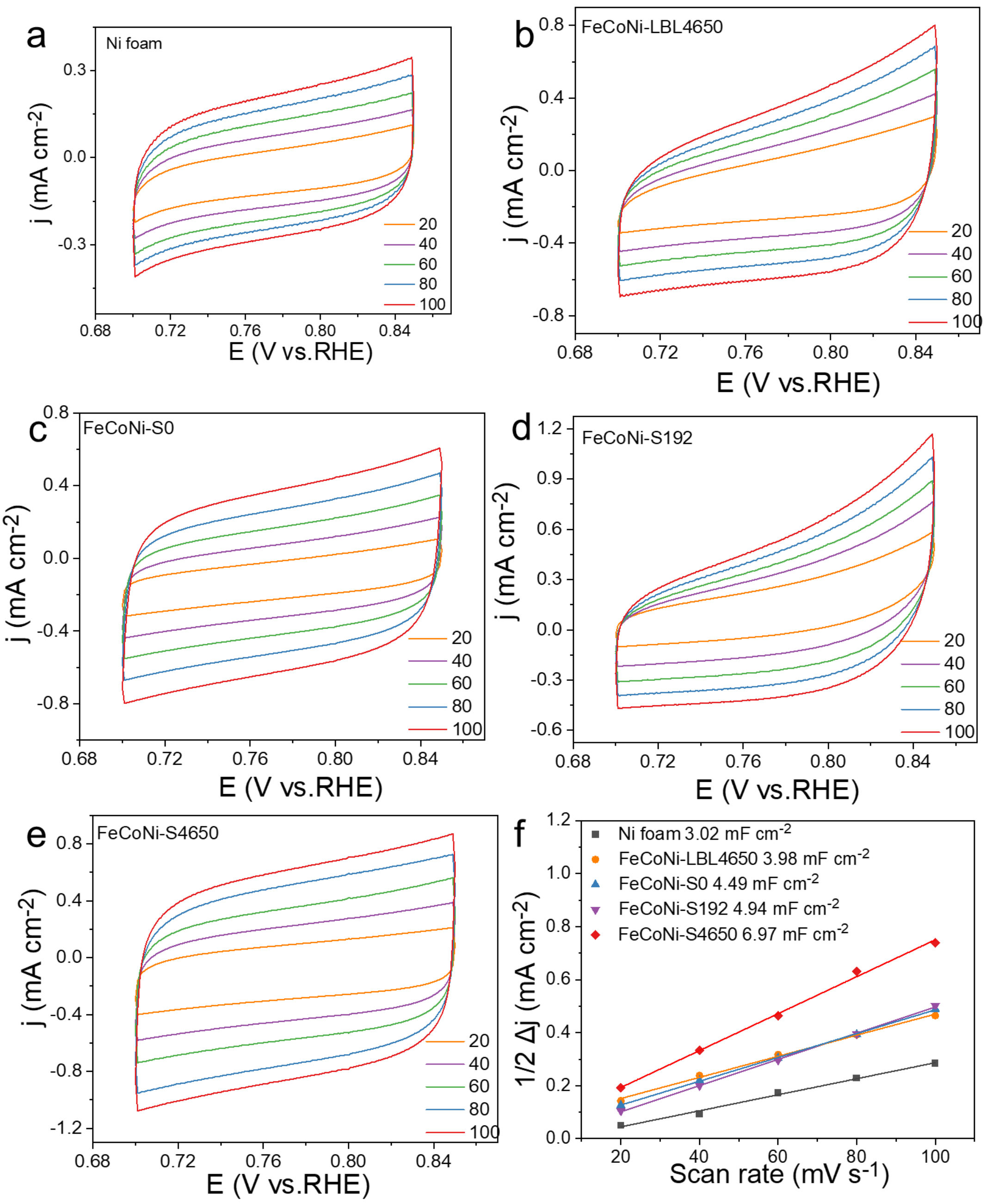

Figure S19. Cyclic voltammetry (CV) cycles at different scan rates at 20, 40, 60, 80 to 100 mV s^{-1} of Ni foam (a), FeCoNi-LBL4650 (b), FeCoNi-S0 (c), FeCoNi-S192 (d), and FeCoNi-S4650 (e). (f) The corresponding current density differences vs. scan rates of the samples to calculate electrochemical double-layer capacitance (C_{dl}). The Cdl is proportional to the electrochemical surface area (ECSA).

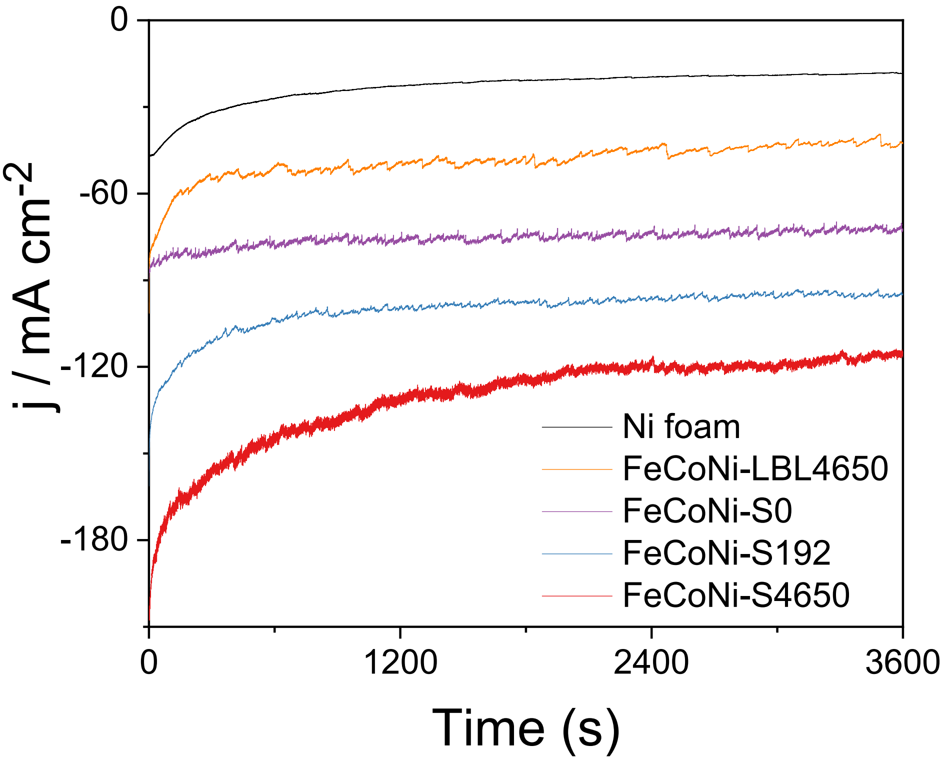

Figure S20. The chronoamperometric profiles of Ni foam, FeCoNi-LBL4650, FeCoNi-S0, FeCoNi-S192, and FeCoNi-S4650 in an electrolyte of 0.5 M Na_2SO_4 and 0.1 M $NaNO_3$ at a fixed potential of −0.7 V vs. RHE.

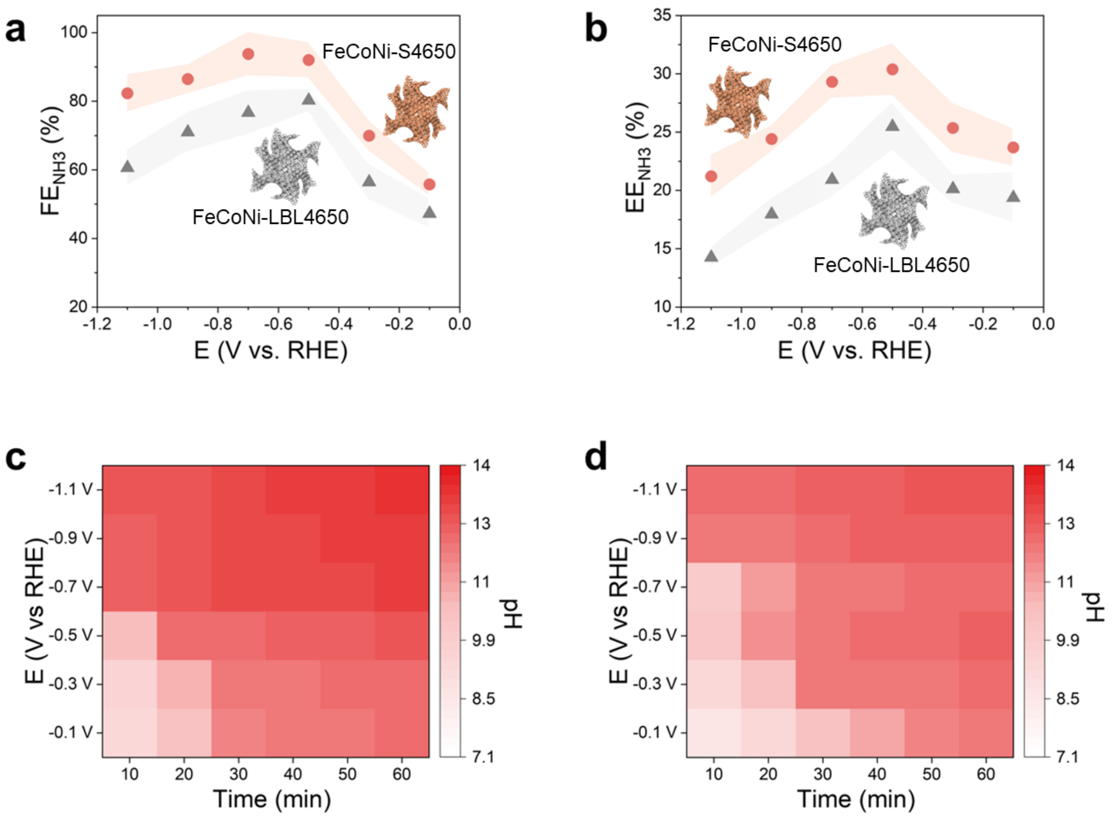

Figure S21. FE $_{NH3}$ (a) and EE_{NH3} (b) of FeCoNi-S4650 and FeCoNi-LBL4650 at varied initial potentials in an electrolyte of 0.5 M Na_2SO_4 and 0.1 M $NaNO_3$. Error bars represent the standard error of the mean derived from three independent replicate measurements. Time-dependent pH variation of FeCoNi-S4650 (c) and FeCoNi-LBL4650 (d) at varied potentials.

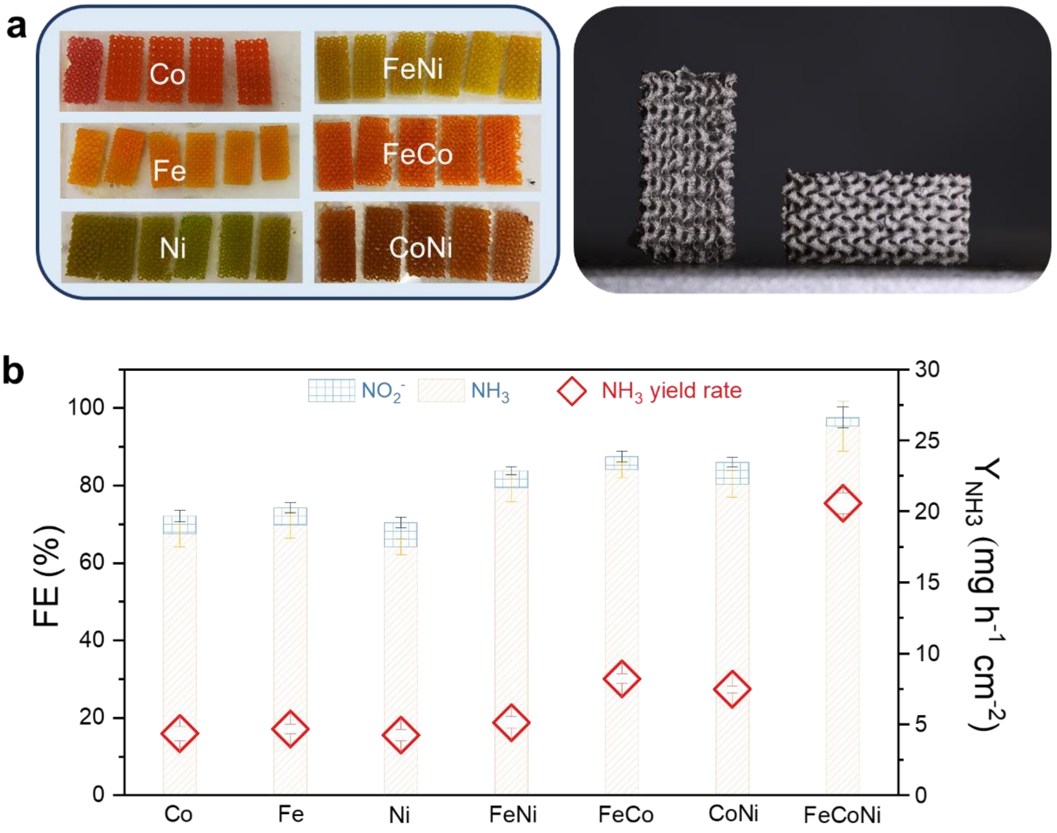

Figure S22. (a) The fabrication of reference samples with different compositional elements. (b) FE_{NH3} and Y_{NH3} of Fe, Co, Ni, FeCo, FeNi, and CoNi, and FeCoNi metamaterial catalysts. Error bars represent the standard error of the mean derived from three independent replicate measurements.

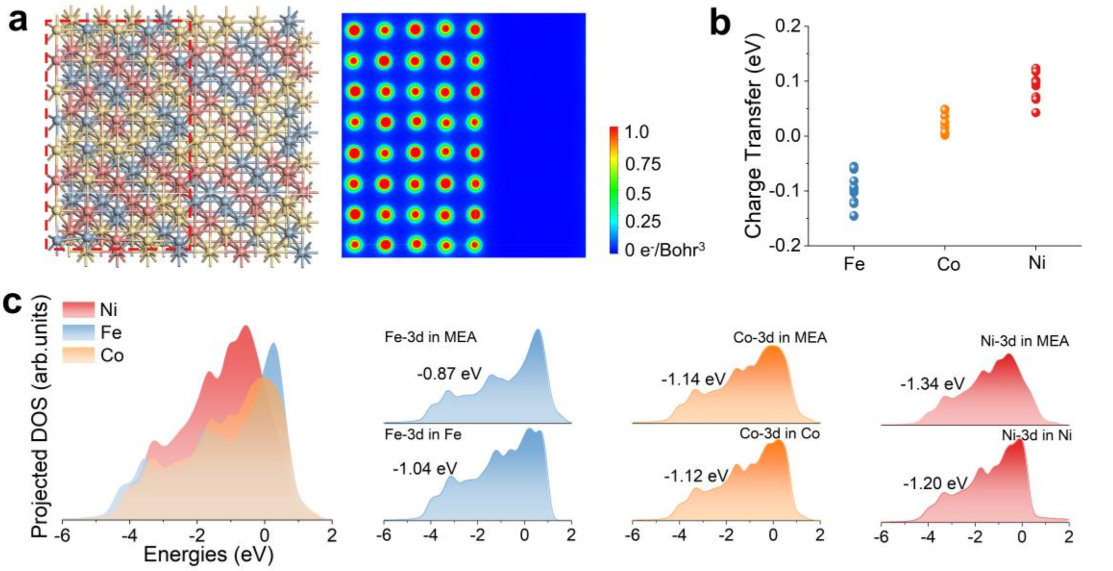

Figure S23. (a) Electron density of the FeCoNi MEA. (b) Bader charges analysis of the FeCoNi MEA. (c) A comparison of computed PDOS of each element in the MEA and pure metals (Fe, Co, and Ni).

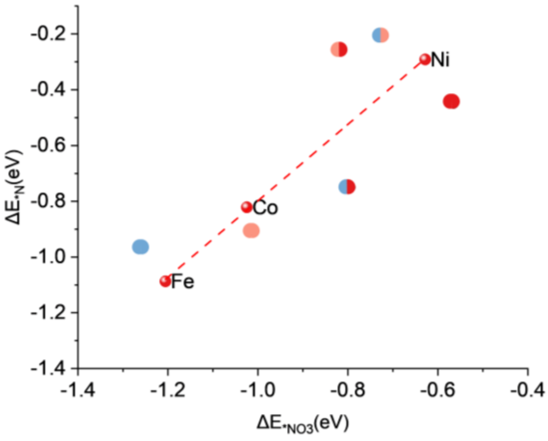

Figure S24. DFT-calculated adsorption energies of $*NO_3$ and *N on pure metal (red ball) and various binding active sites in the MEA (Fe: blue; Co: yellow; Ni: red).

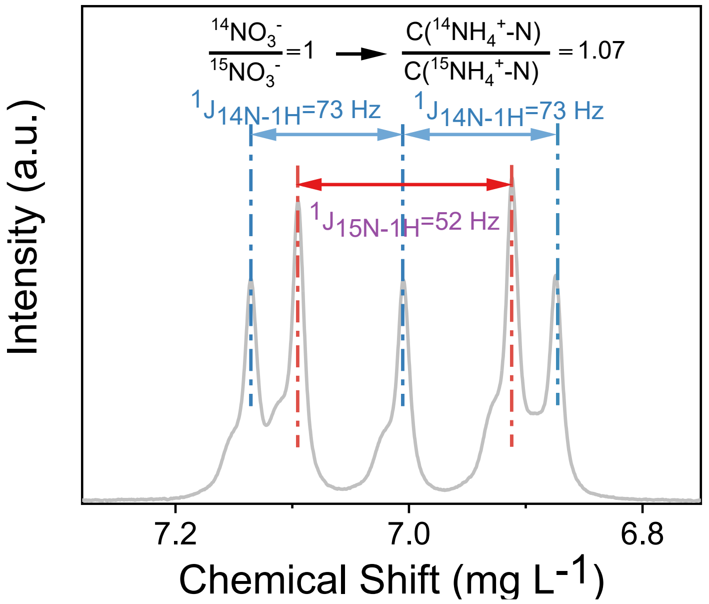

Figure S25. ^1H NMR spectra for the electrolytes after nitrate reduction test with $^{14}NO_3^-$ /$^{15}NO_3^-$ in a ratio of 1 to 1 as feed.

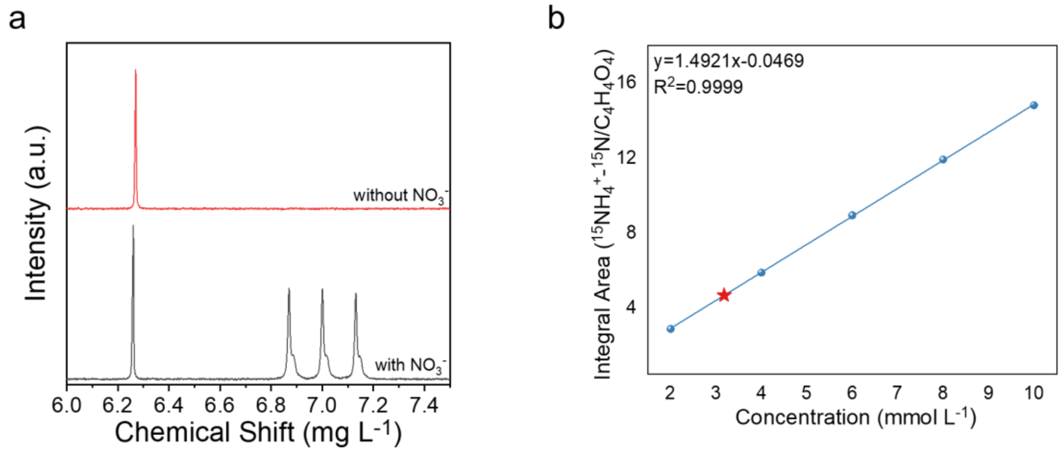

Figure S26. (a) The 1H NMR spectra of the electrolyte after NO_3RR electrolysis with and without $^{15}NO_3^-$ as the original N sources. (b) The standard curve of integral area ($^{14}(NH_4)_2SO_4/C_4H_4O_4$) against $^{14}(NH_4)_2SO_4$ concentration.

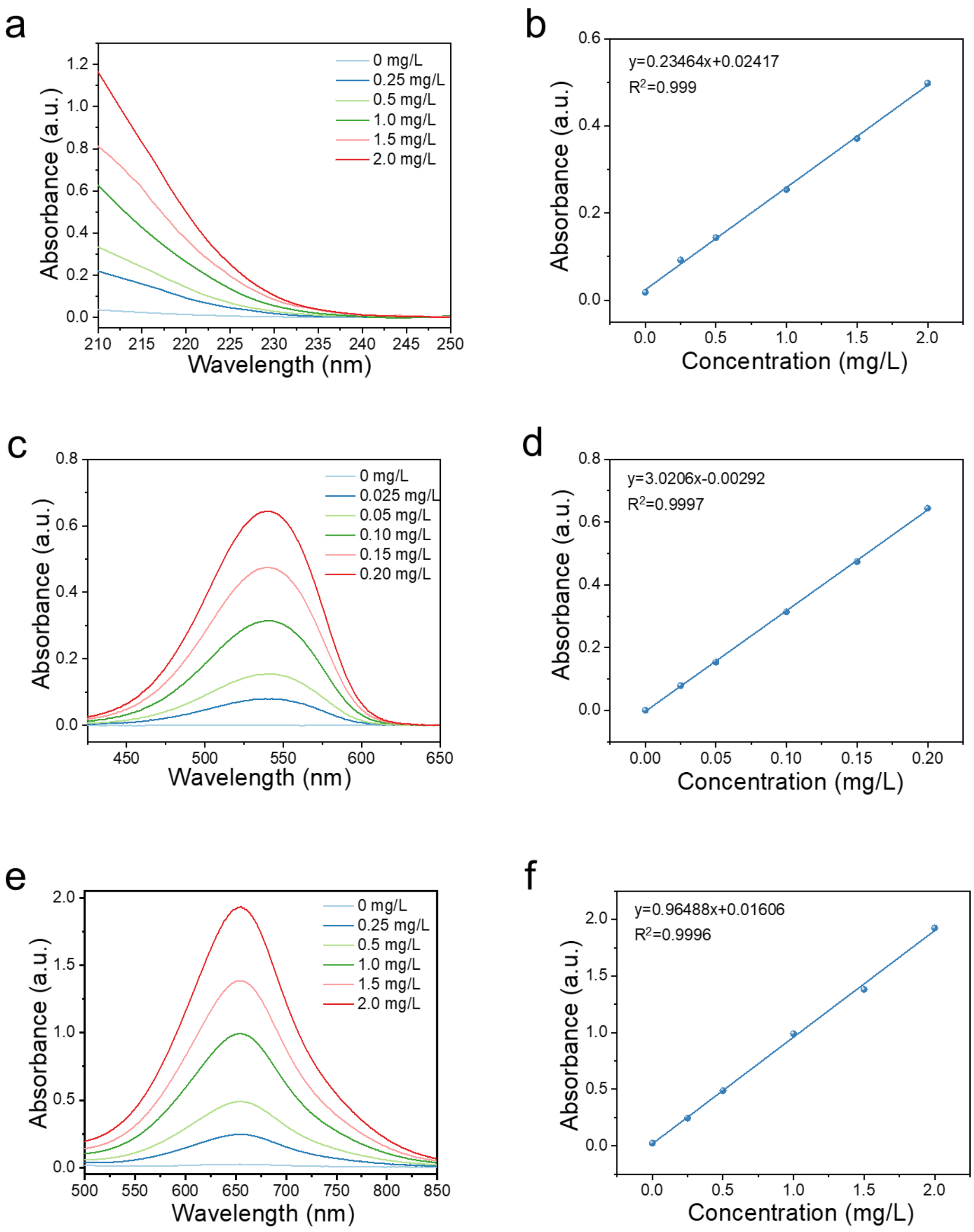

Figure S27. (a) UV-Vis absorbance of a series of standard concentrations of KNO_3 solutions. (b) The fitted concentration-absorbance curve for determining the concentration of NO_3^-. (c) UV-Vis absorbance curves of the Griess method using a series of standard concentration of KNO_2 solutions. (d) The fitted concentration-absorbance curve for determining the concentration of NO_2^-. (e) UV-Vis absorbance curves of the indophenol blue method using a series of standard concentration of $NH_4(SO_4)_2$ solutions. (f) The fitted concentration-absorbance curve for determining the concentration of NH_4^+.

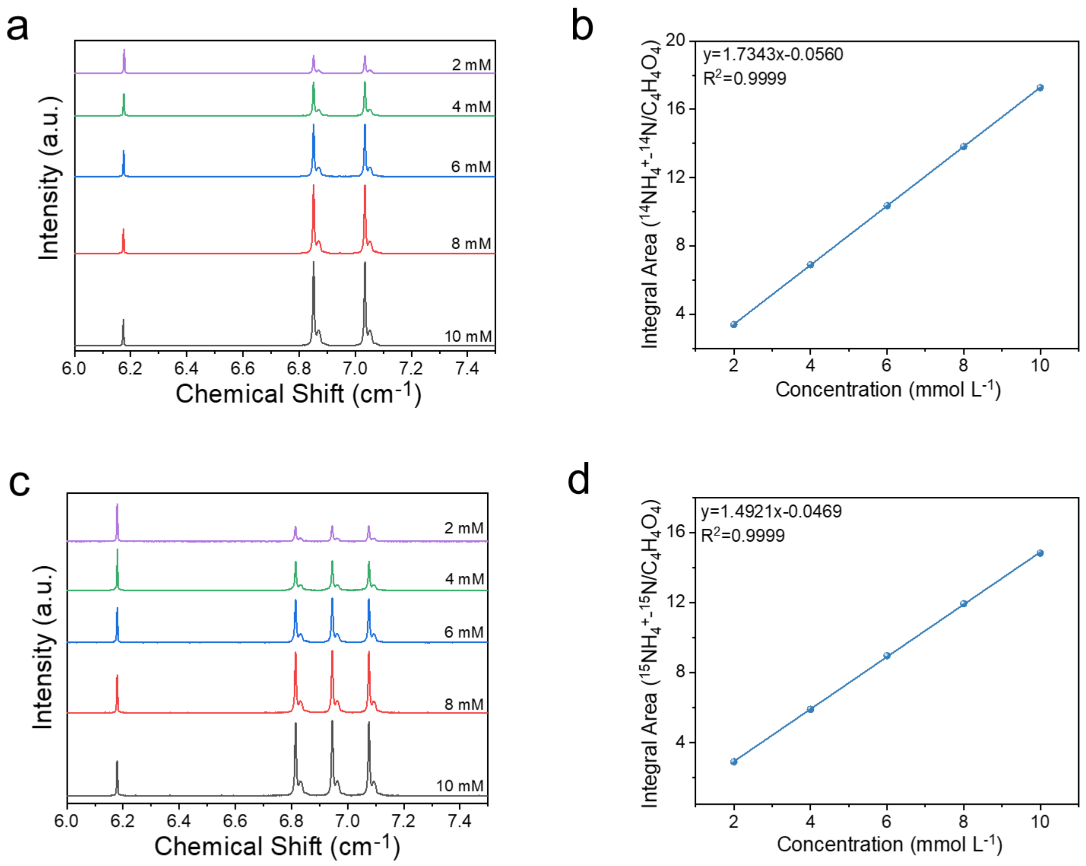

Figure S28. The 1H NMR spectra of $^{14}NH_4^+$-^{14}N with $^{14}(NH_4)_2SO_4$ and $C_4H_4O_4$ as standards. b The standard curve of integral area ($^{14}(NH_4)_2SO_4/C_4H_4O_4$) against $^{14}(NH_4)_2SO_4$ concentration. c The 1H NMR spectra of $^{15}NH_4^+$-^{15}N with $^{15}(NH_4)_2SO_4$ and $C_4H_4O_4$ as standards. d The standard curve of integral area ($^{15}(NH_4)_2SO_4/C_4H_4O_4$) against $^{15}(NH_4)_2SO_4$ concentration.

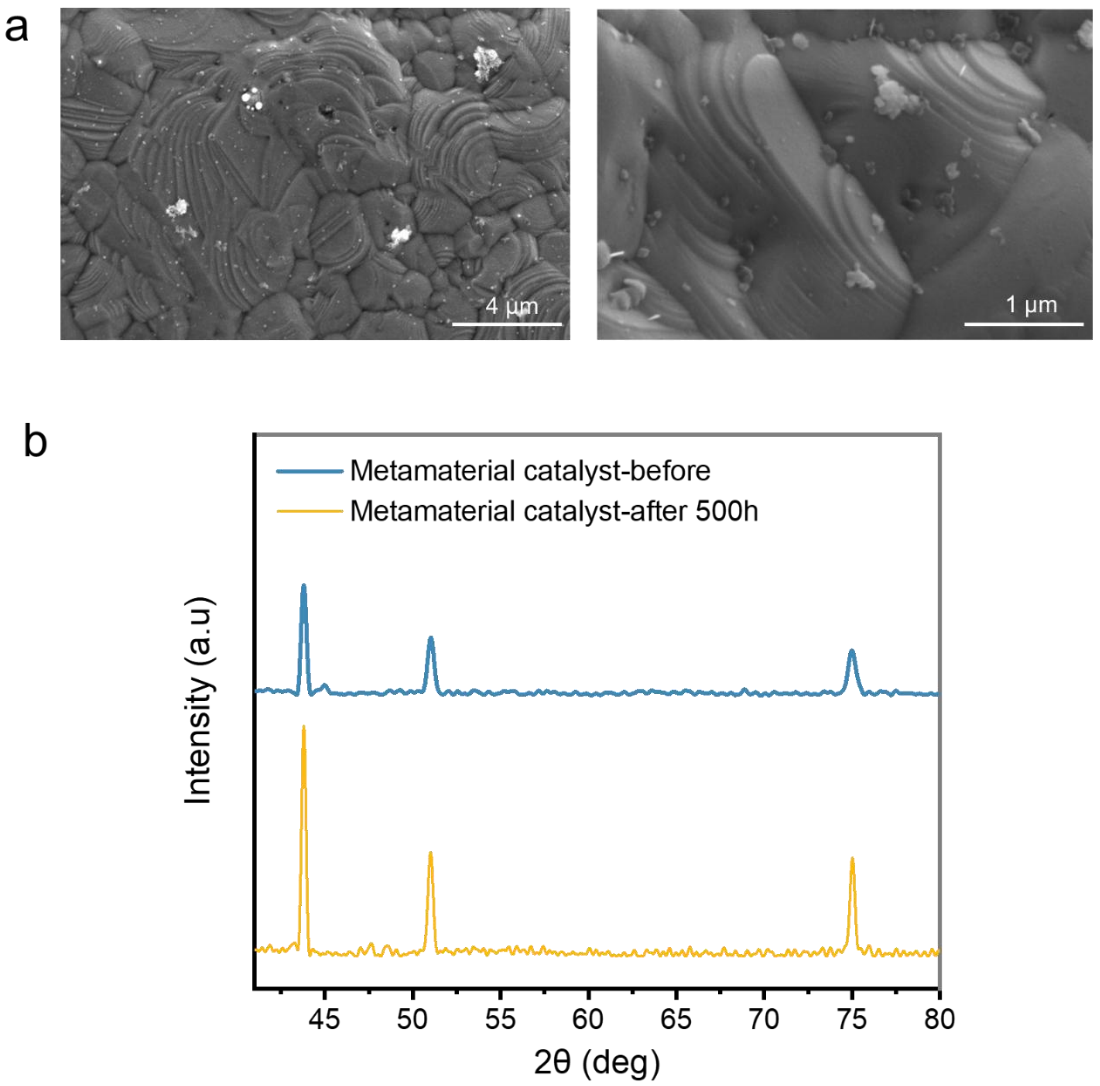

Figure S29. (a) SEM images of FeCoNi-S4650 electrode after long-term stability test. (b) XRD of FeCoNi-S4650 electrode before and after long-term stability test.

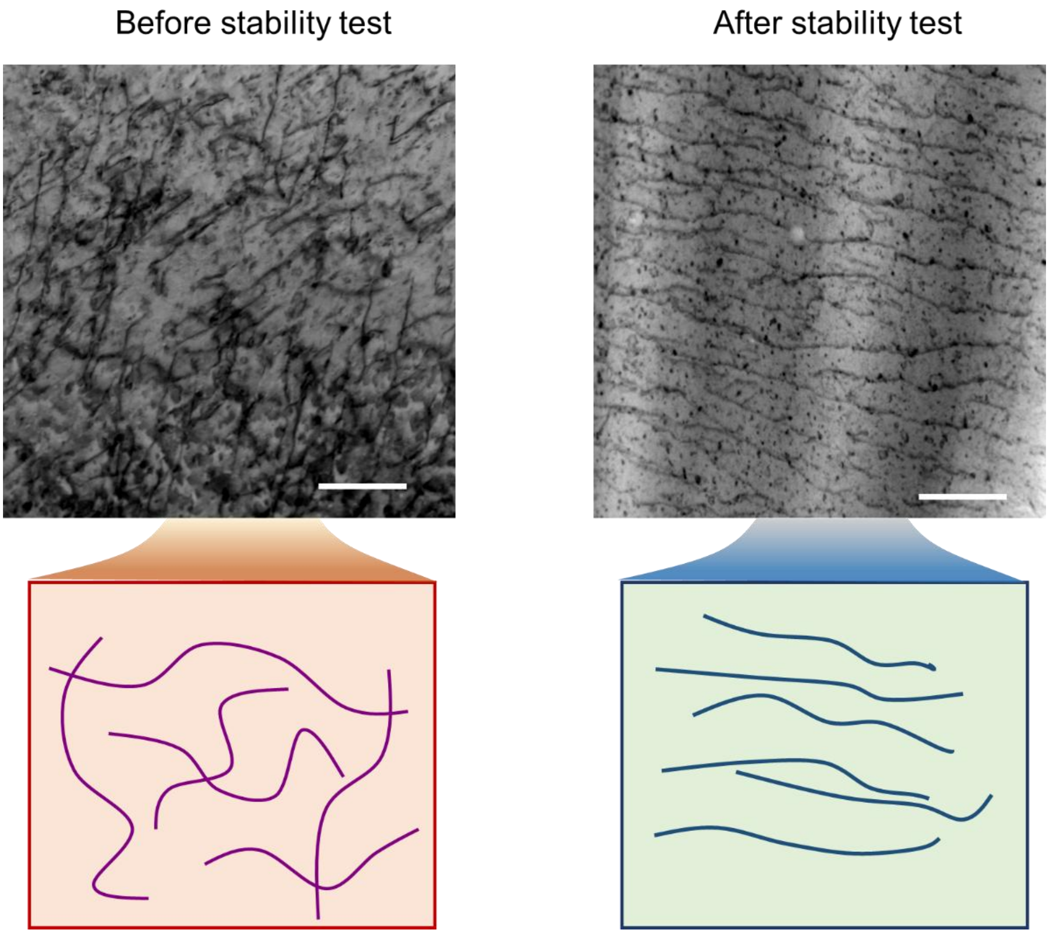

Figure S30. The comparison of dislocation morphology before and after catalytic stability test. Scale bar: 100 nm.

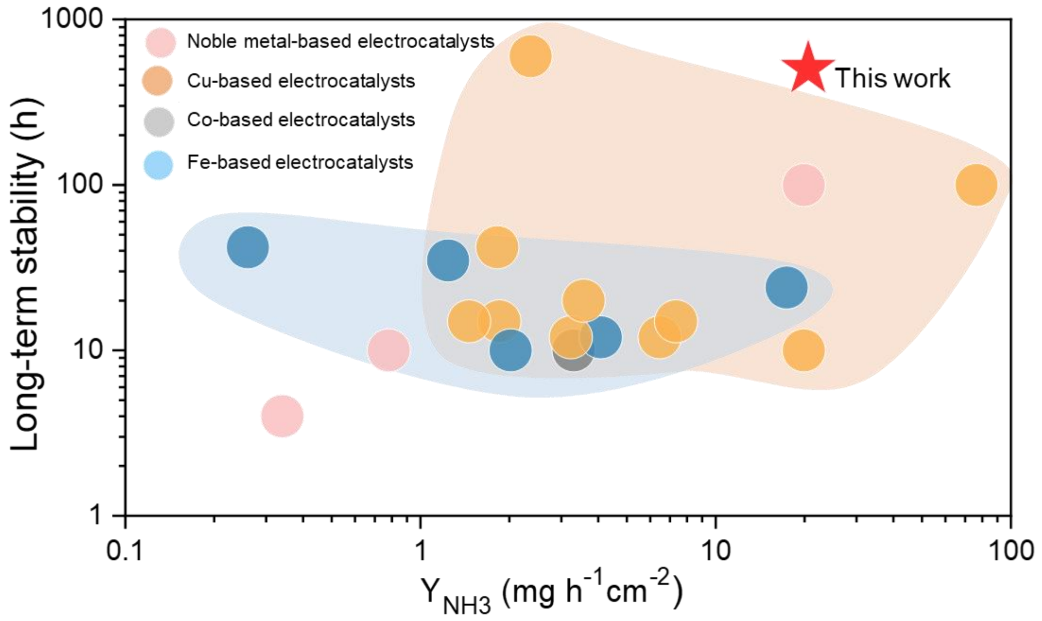

Figure S31. Comparison of NH_3 yield rates and long-term stability of the reported electrocatalysts for NO_3^--to-NH_3 conversion.

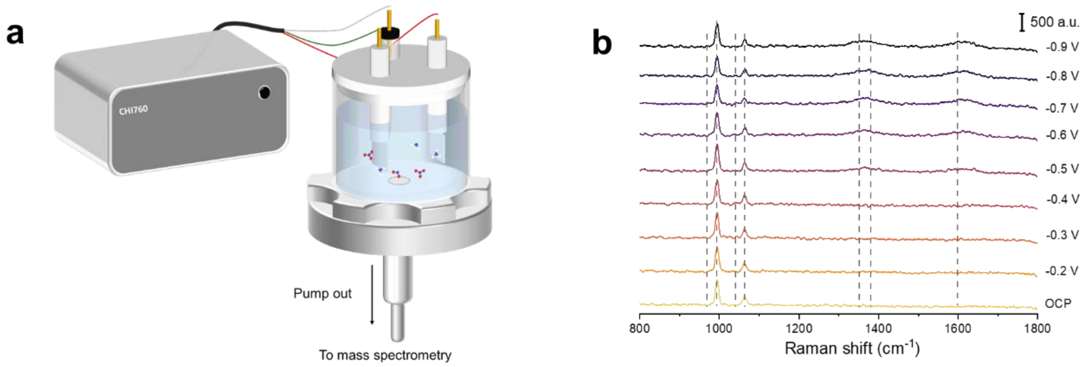

Figure S32. (a) The schematic of the DEMS electrochemical cell. (b) *in situ* Raman spectra of NO_3RR over FeCoNi-LBL at different applied potentials in 0.5 M Na_2SO_4 and 0.1 M $NaNO_3$.

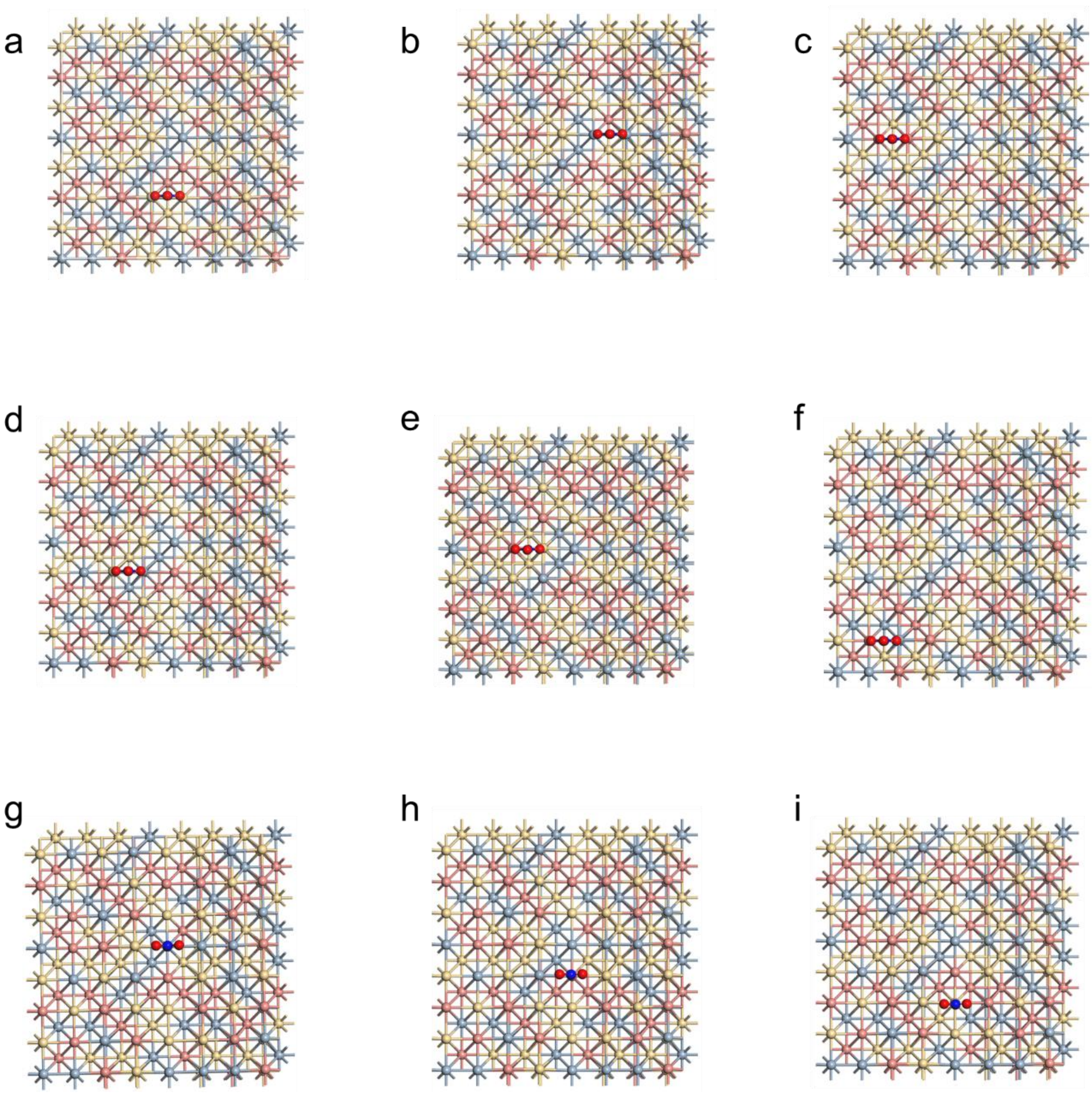

Figure S33. The DFT determined active sites with ΔG_{*NO3} values on the various binding active sites Ni-Ni (a), Fe-Fe (b), CoCo (c), FeNi (d), CoNi (e), FeCo (f), Fe (g), Co (h), Ni (i) in FeCoNi-LBL slab surface.

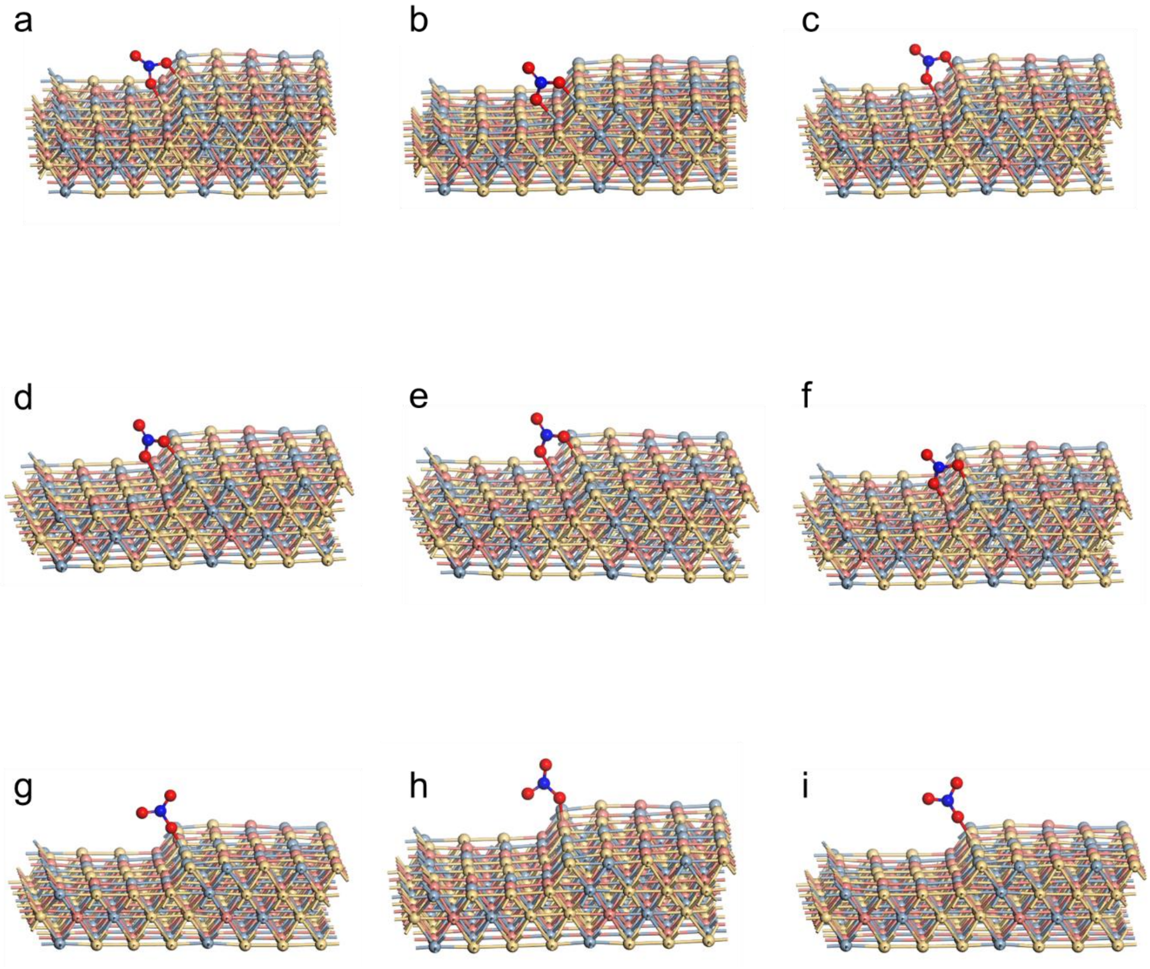

Figure S34. The DFT determined active sites with ΔG_{*NO3} values on the various binding active sites Ni-Ni (a), Fe-Fe (b), CoCo (c), FeNi (d), CoNi (e), FeCo (f), fe (g), Co (h), Ni (i) in the stepped FeCoNi slab surface.

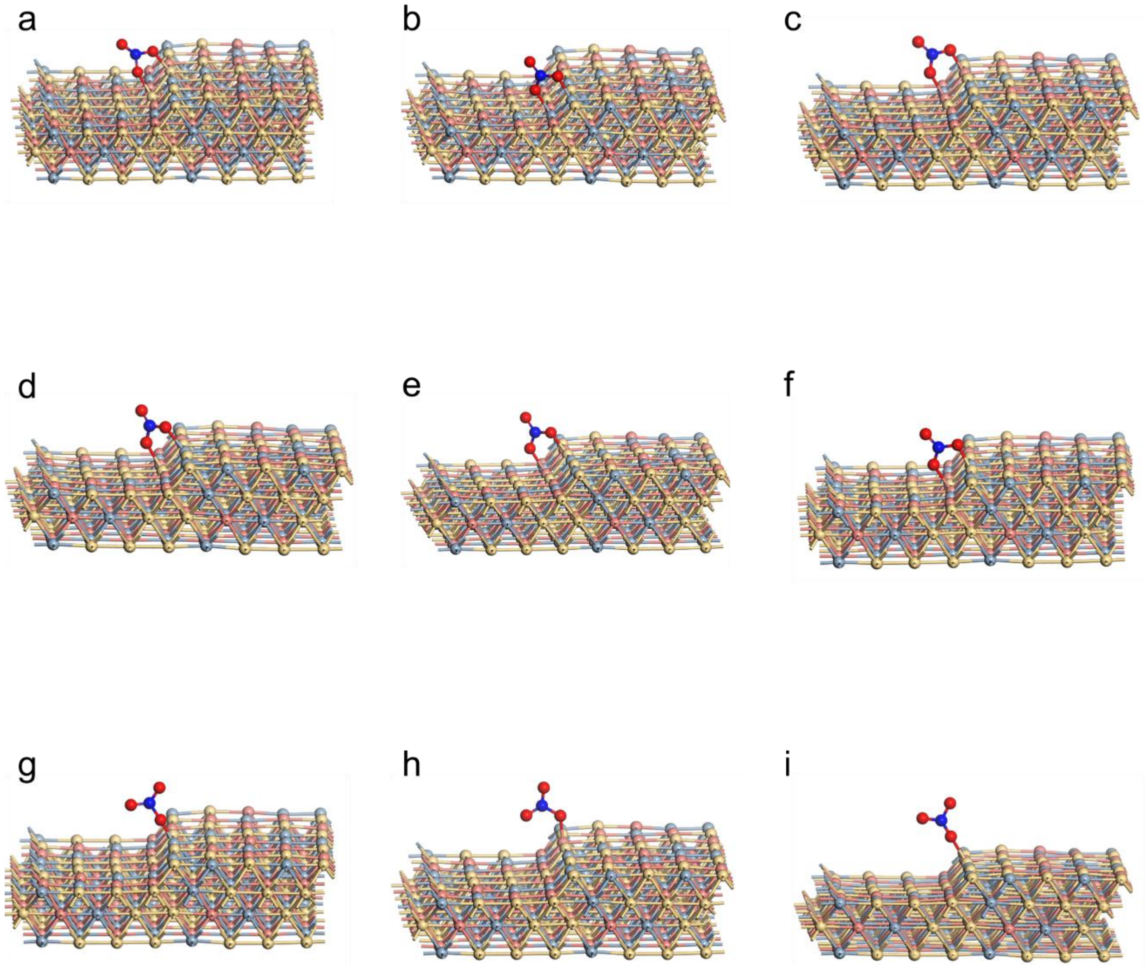

Figure S35. The DFT determined active sites with ΔG_{*NO3} values on the various binding active sites Ni-Ni (a), Fe-Fe (b), CoCo (c), FeNi (d), CoNi (e), FeCo (f), fe (g), Co (h), Ni (i) in the stepped FeCoNi with 3% strain slab surface.

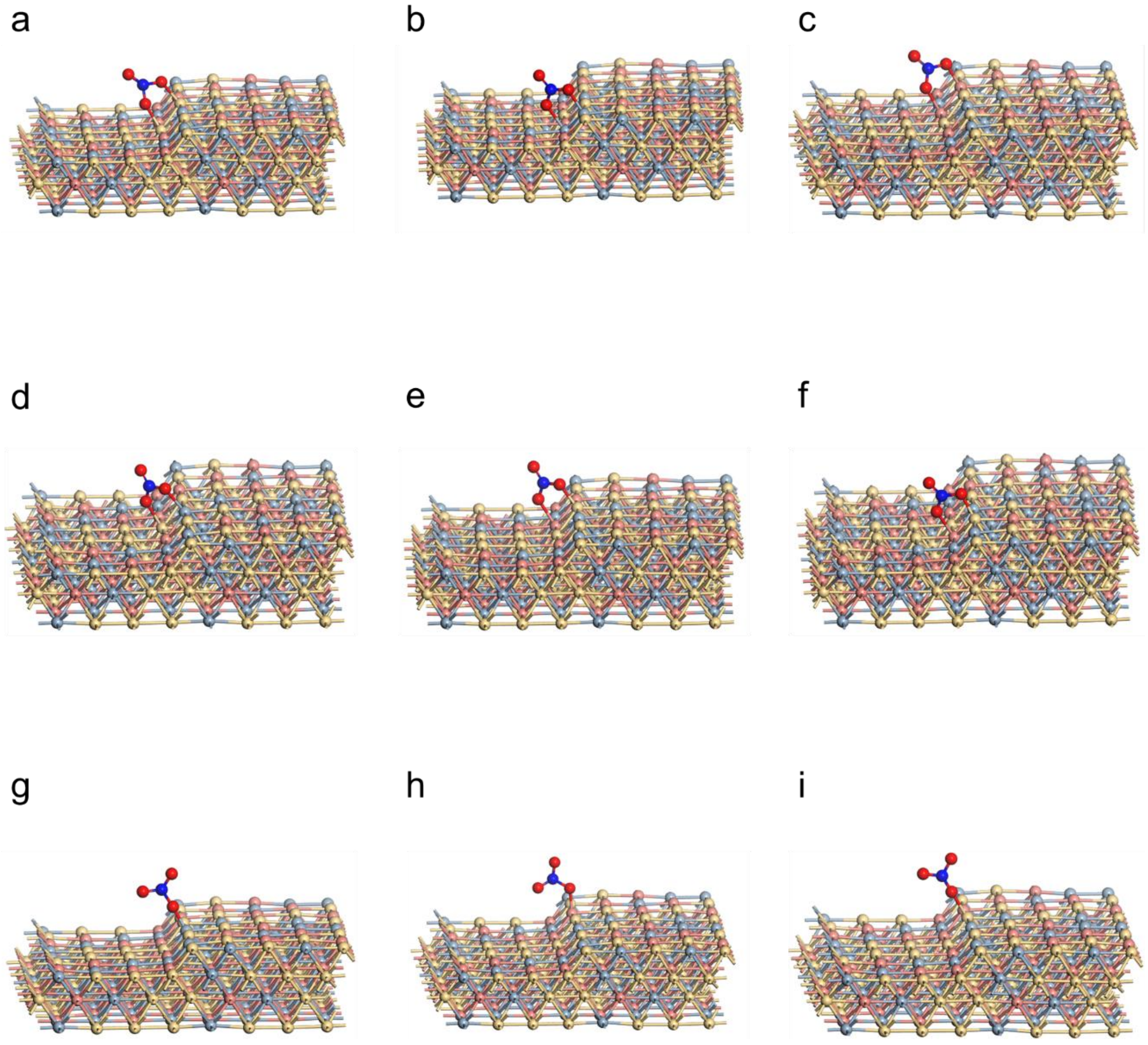

Figure S36. The DFT determined active sites with ΔG_{*NO3} values on the various binding active sites Ni-Ni (a), Fe-Fe (b), CoCo (c), FeNi (d), CoNi (e), FeCo (f), fe (g), Co (h), Ni (i) in the stepped FeCoNi with 6% strain slab surface.

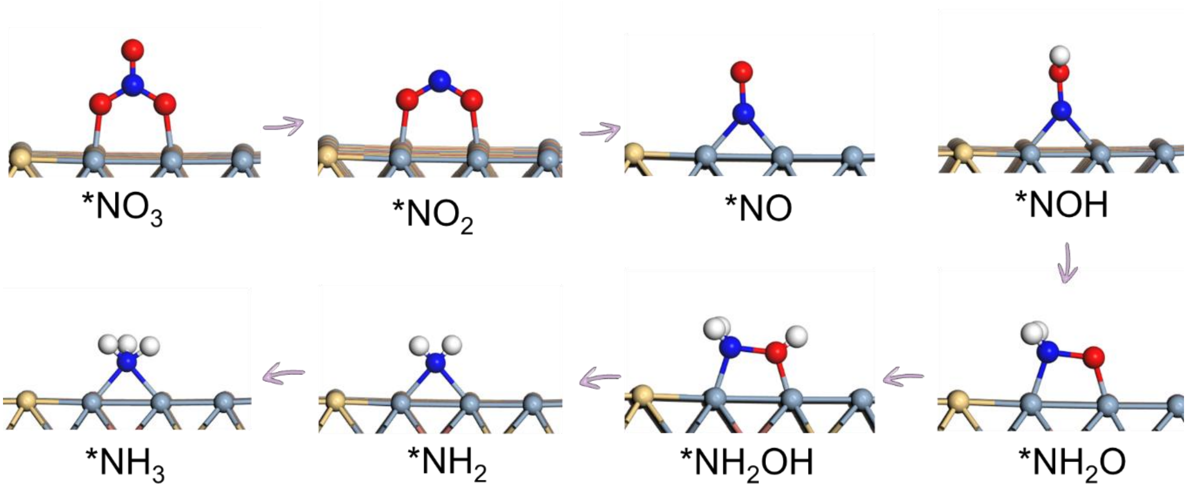

Figure S37. The adsorption models of various intermediates generated during NO_3RR pathways on the FeCoNi-LBL slab surface.

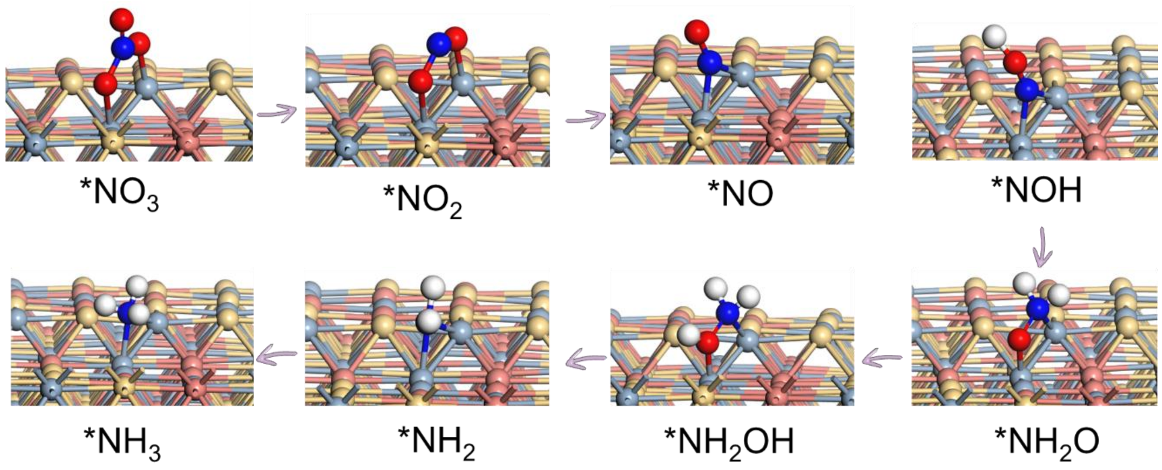

Figure S38. The adsorption models of various intermediates generated during NO_3RR pathways on the stepped FeCoNi slab surface.

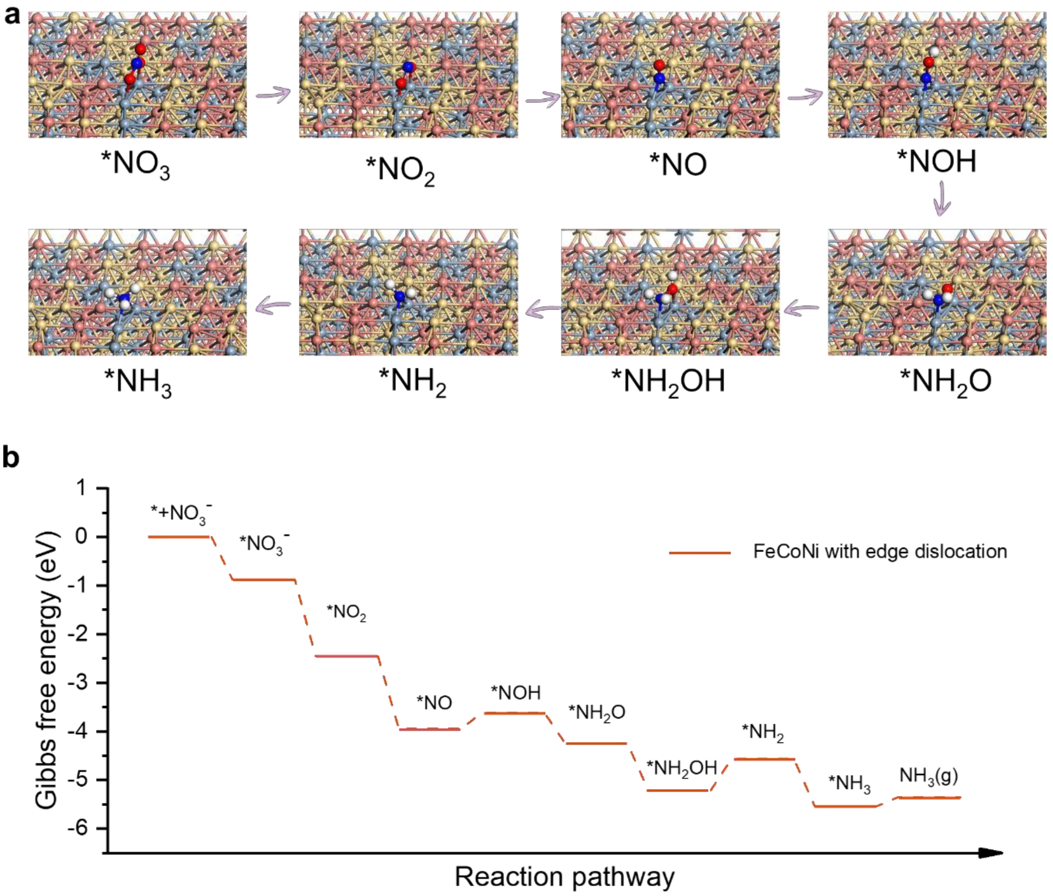

Figure S39. (a) The adsorption models of various intermediates generated during NO_3RR pathways on the surface of FeCoNi slab edge dislocations. (b) Free-energy diagram for NO_3RR on FeCoNi slab edge dislocations.

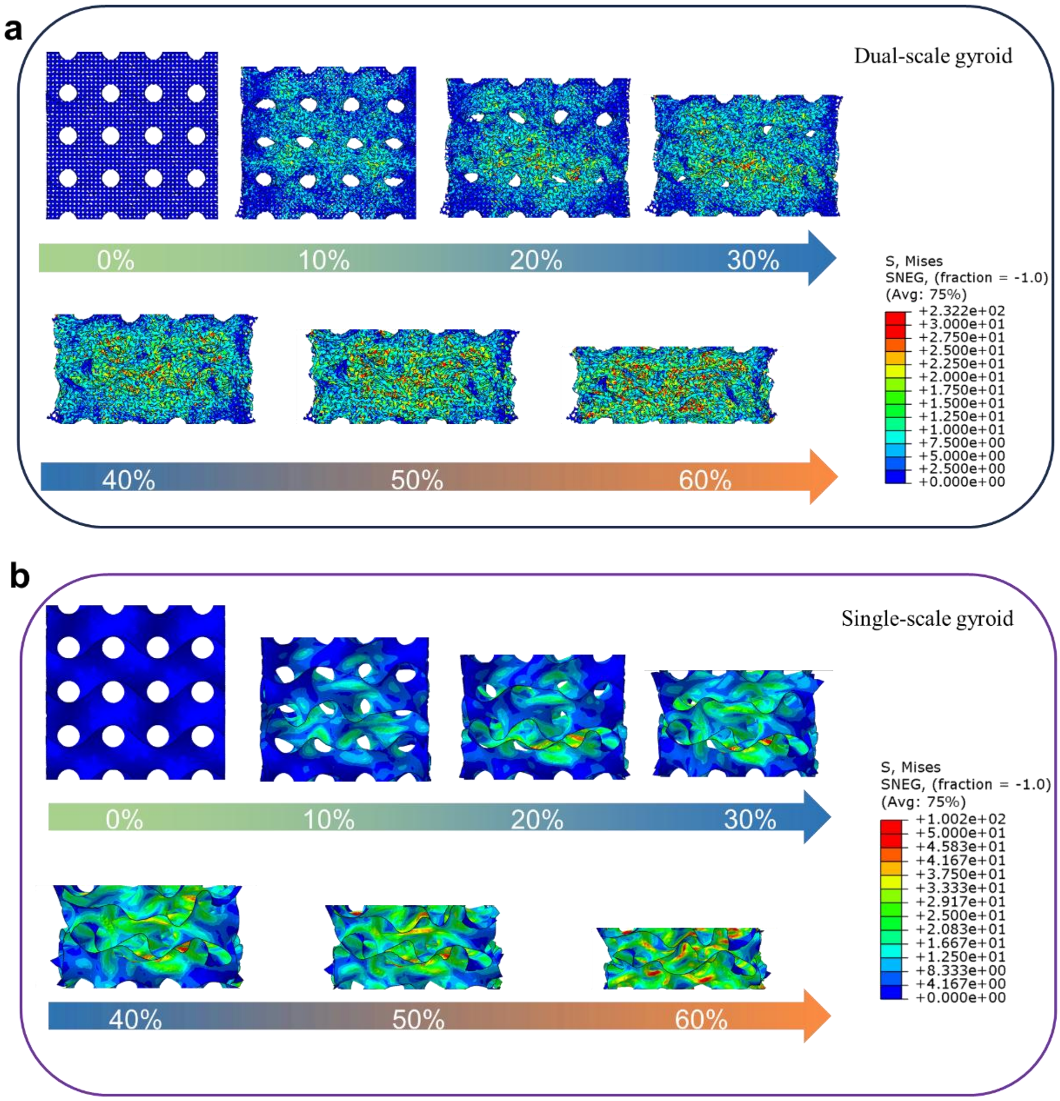

Figure S40. Finite element simulation results of FeCoNi (a) dual-scale gyroid and (b) single-scale gyroid at different compression strains at a constant strain rate of 1.0×10^{-3} s^{-1}.

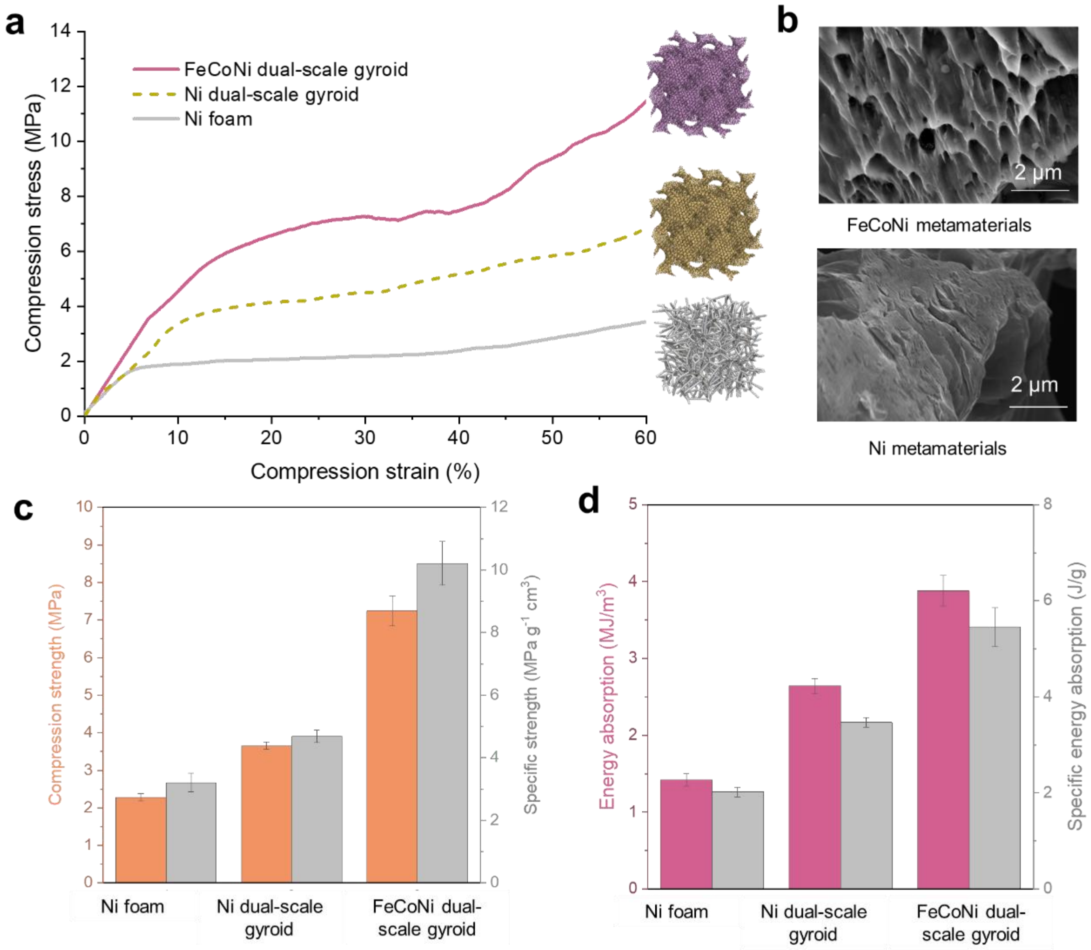

Figure S41. Mechanical properties and deformation microstructure of FeCoNi dual-scale gyroid, Ni dual-scale gyroid, and Ni foam. (a) Compression stress-strain curve of different samples with an equivalent relative density of ~7%. (b) Comparison of fracture surface morphologies. (c-d) Comparison of (c) compression strength/ specific compression strength and (d) energy absorption/ specific energy absorption. The error bars indicate the standard deviations calculated from three replicate measurements.

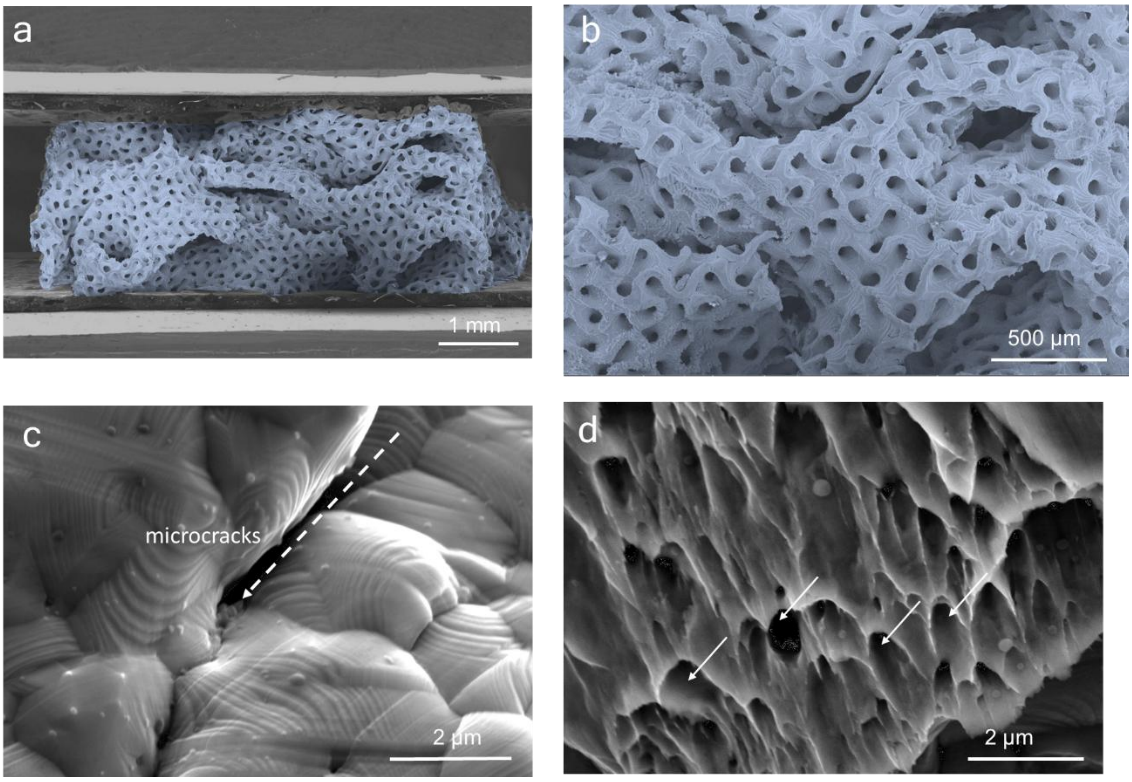

Figure S42. The morphology and microstructure of post-deformation sample. (a-b) SEM image revealing the densely packed deformation behavior of thin walls with very few cracks. (c)SEM image displaying the pathway of microcracks. (d) SEM image displaying the numerous dimples at the fracture region.

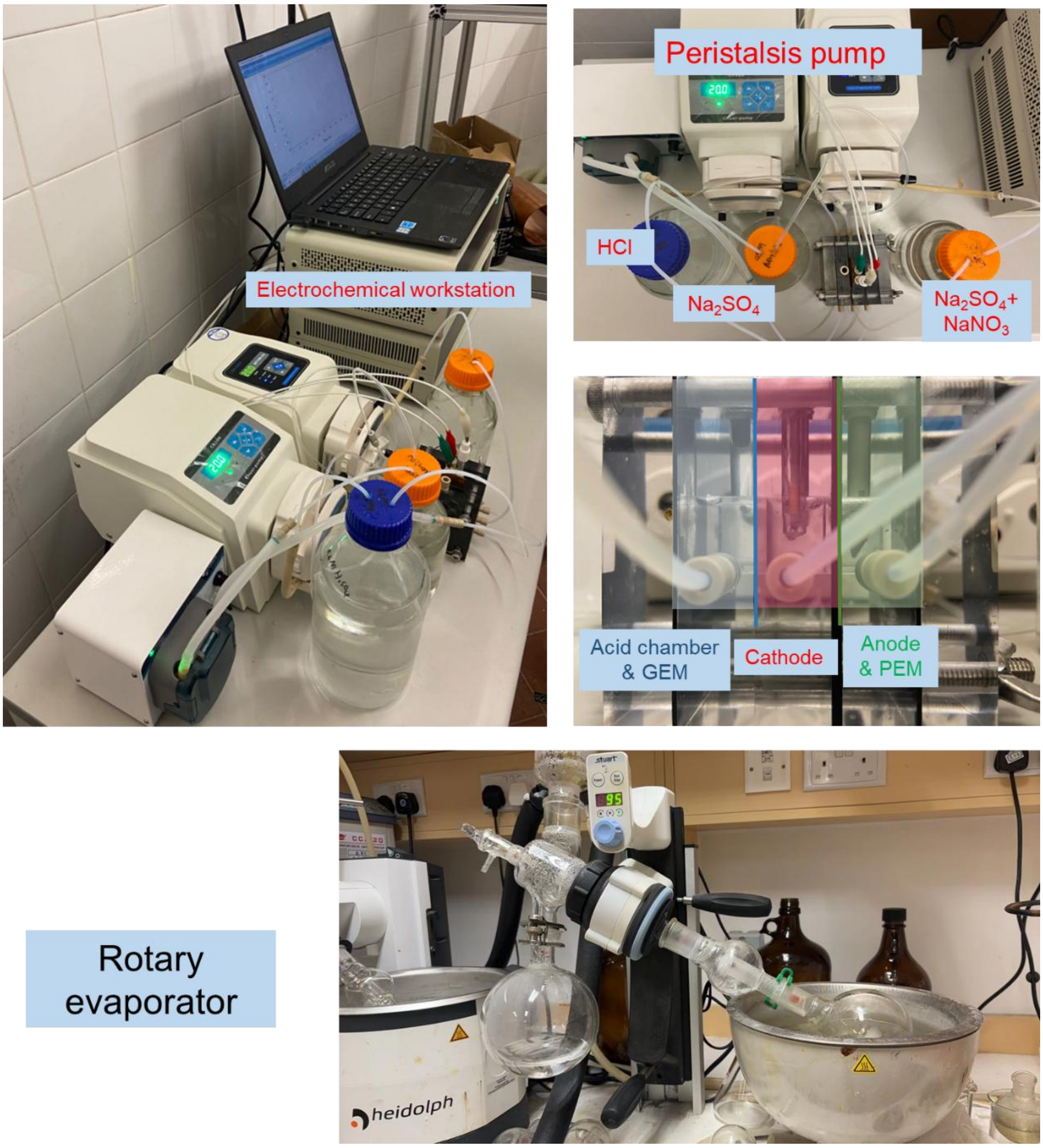

Figure S43. Reactor for conducting long-term NO_3RR conversion experiments and the rotary evaporator for collecting NH_4Cl products. Three-chamber reactor consists of an anode chamber (20x20x10mm^3), a cathode chamber (20x20x10mm^3), and an acid absorption chamber (20x20x10mm^3). The immersed areas of metamaterial catalyst were 1 cm^2 (1 cm × 1 cm).

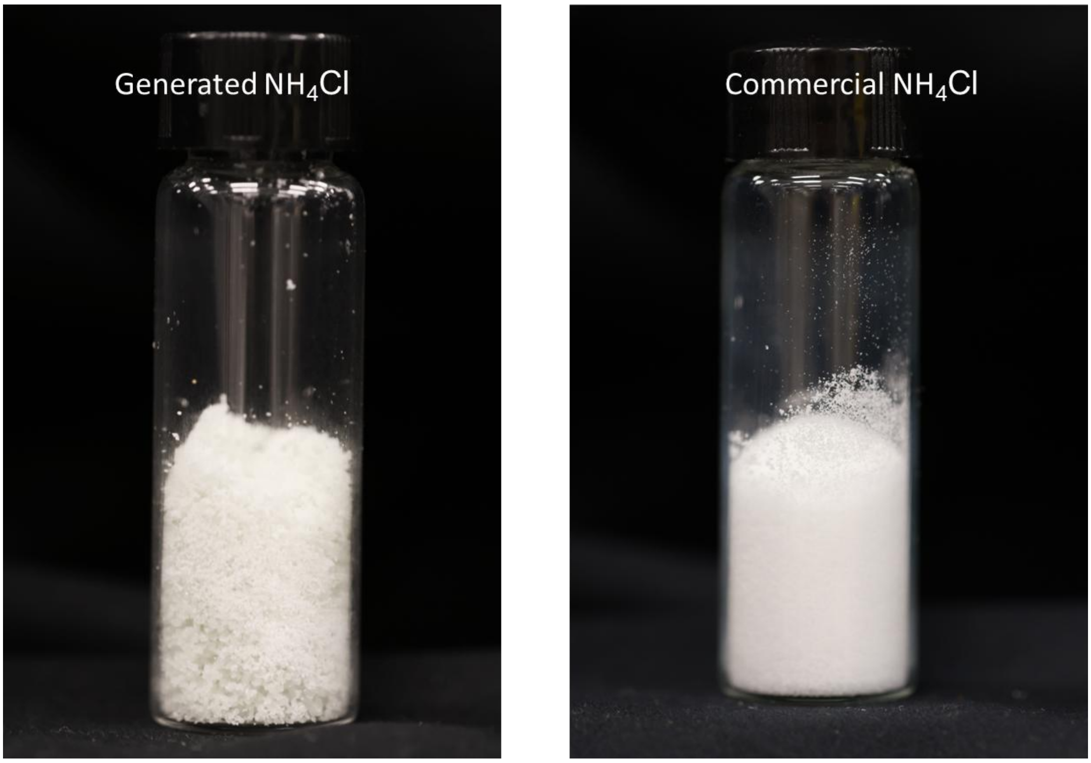

Figure S44. Photo displaying the comparison of generated NH_4Cl and commercial NH_4Cl product.

References

1 Meng, F., Morin, S. A., Forticaux, A. & Jin, S. Screw dislocation driven growth of nanomaterials. *Accounts of chemical research* **46**, 1616-1626 (2013).

2 Meng, X. *et al.* Hierarchical triphase diffusion photoelectrodes for photoelectrochemical gas/liquid flow conversion. *Nature Communications* **14**, 2643 (2023).

3 Persson, A. I. *et al.* Solid-phase diffusion mechanism for GaAs nanowire growth. *Nature materials* **3**, 677-681 (2004).

4 Markov, I. V. *Crystal growth for beginners: fundamentals of nucleation, crystal growth and epitaxy*. (World scientific, 2016).

5 Morin, S. A., Bierman, M. J., Tong, J. & Jin, S. Mechanism and kinetics of spontaneous nanotube growth driven by screw dislocations. *Science* **328**, 476-480 (2010).

6 Chu, Y., Jing, S., Liu, D., Liu, J. & Zhao, Y. Morphological control and kinetics in three dimensions for hierarchical nanostructures growth by screw dislocations. *Acta Materialia* **162**, 284-291 (2019).

7 Simsek, U., Gayir, C. E., Kiziltas, G. & Sendur, P. An integrated homogenization–based topology optimization via RBF mapping strategies for additively manufactured FGLS and its application to bandgap structures. *The International Journal of Advanced Manufacturing Technology* **111**, 1361-1374 (2020).

8 Zhang, S. *et al.* Fe/Cu diatomic catalysts for electrochemical nitrate reduction to ammonia. *Nature Communications* **14**, 3634 (2023).

9 Wei, M. *et al.* High-entropy alloy nanocrystal assembled by nanosheets with d–d electron interaction for hydrogen evolution reaction. *Energy & Environmental Science* **16**, 4009-4019 (2023).

10 Gao, Q. *et al.* Breaking adsorption-energy scaling limitations of electrocatalytic nitrate reduction on intermetallic CuPd nanocubes by machine-learned insights. *Nature communications* **13**, 2338 (2022).

11 Sun, S., Zhou, X., Cong, B., Hong, W. & Chen, G. Tailoring the d-band centers endows (Ni x Fe1–x) 2P nanosheets with efficient oxygen evolution catalysis. *Acs Catalysis* **10**, 9086-9097 (2020).

12 Wang, S., Pillai, H. S. & Xin, H. Bayesian learning of chemisorption for bridging the complexity of electronic descriptors. *Nature communications* **11**, 6132 (2020).

13 Chen, Z. W. *et al.* Unusual Sabatier principle on high entropy alloy catalysts for hydrogen evolution reactions. *Nature Communications* **15**, 359 (2024).

14 Fang, J.-Y. *et al.* Ampere-level current density ammonia electrochemical synthesis using CuCo nanosheets simulating nitrite reductase bifunctional nature. *Nature Communications* **13**, 7899 (2022).

15 He, Q. & Yang, Y. On lattice distortion in high entropy alloys. *Frontiers in Materials* **5**, 42 (2018).

16 Jin, K., Zhang, C., Zhang, F. & Bei, H. Influence of compositional complexity on interdiffusion in Ni-containing concentrated solid-solution alloys. *Materials Research Letters* **6**, 293-299 (2018).

17 Hÿtch, M., Snoeck, E. & Kilaas, R. Quantitative measurement of displacement and strain fields from HREM micrographs. *Ultramicroscopy* **74**, 131-146 (1998).

18 Qu, S., Ding, J., Fu, J., Fu, M. & Song, X. Anisotropic material properties of pure copper with fine-grained microstructure fabricated by laser powder bed fusion process. *Additive Manufacturing* **59**, 103082 (2022).

19 Thirathipviwat, P. *et al.* A comparison study of dislocation density, recrystallization and grain growth among nickel, FeNiCo ternary alloy and FeNiCoCrMn high entropy alloy. *Journal of Alloys and Compounds* **790**, 266-273 (2019).

20 Varvenne, C., Luque, A. & Curtin, W. A. Theory of strengthening in fcc high entropy alloys. *Acta Materialia* **118**, 164-176 (2016).

21 Wang, Y. *et al.* Enhanced nitrate-to-ammonia activity on copper–nickel alloys via tuning of intermediate adsorption. *Journal of the American Chemical Society* **142**, 5702-5708 (2020).

22 Niu, H. *et al.* Theoretical insights into the mechanism of selective nitrate-to-ammonia electroreduction on single-atom catalysts. *Advanced Functional Materials* **31**, 2008533 (2021).

23 John, J., MacFarlane, D. R. & Simonov, A. N. The why and how of NO x electroreduction to ammonia. *Nature Catalysis* **6**, 1125-1130 (2023).

24 Zeng, Y., Priest, C., Wang, G. & Wu, G. Restoring the nitrogen cycle by electrochemical reduction of nitrate: progress and prospects. *Small Methods* **4**, 2000672 (2020).

25 Han, S. *et al.* Ultralow overpotential nitrate reduction to ammonia via a three-step relay mechanism. *Nature catalysis* **6**, 402-414 (2023).

26 Zhang, Y. *et al.* Dislocation-engineered piezocatalytic water splitting in single-crystal BaTiO 3. *Energy & Environmental Science* **18**, 602-612 (2025).

27 Hao, S., Edalati, K., Gao, Q. & Lin, H.-J. Effect of crystal defects on the electrocatalytic CO2 reduction performance of pure copper. *Scripta Materialia* **252**, 116268 (2024).

28 Kim, H. J., Mori, K., Nakano, T. & Yamashita, H. Robust Self-Catalytic Reactor for CO2 Methanation Fabricated by Metal 3D Printing and Selective Electrochemical Dissolution. *Advanced Functional Materials* **33**, 2303994 (2023).

29 Han, C. *et al.* Hydrogen Spillover Enabled by Edge Dislocations for Efficient Hydrogen Evolution. *Advanced Functional Materials*, 2425615 (2025).

30 Thiagarajan, S., Thaiyan, M. & Ganesan, R. Physical vapor deposited highly oriented V 2 O 5 thin films for electrocatalytic oxidation of hydrazine. *RSC advances* **6**, 82581-82590 (2016).

31 Zhou, M. *et al.* Dislocation Network-Boosted PtNi Nanocatalysts Welded on Nickel Foam for Efficient and Durable Hydrogen Evolution at Ultrahigh Current Densities. *Advanced Energy Materials* **13**, 2202595 (2023).

32 Gao, W. *et al.* Alloying of Cu with Ru enabling the relay catalysis for reduction of nitrate to ammonia. *Advanced Materials* **35**, 2202952 (2023).

33 Gu, Z. *et al.* Intermediates Regulation via Electron-Deficient Cu Sites for Selective Nitrate-to-Ammonia Electroreduction. *Advanced Materials* **35**, 2303107 (2023).

34 Sun, W. J. *et al.* Built-in electric field triggered interfacial accumulation effect for efficient nitrate removal at ultra-low concentration and electroreduction to ammonia. *Angewandte Chemie International Edition* **60**, 22933-22939 (2021).

35 Zhao, R. *et al.* A Bi-Co Corridor Construction Effectively Improving the Selectivity of Electrocatalytic Nitrate Reduction toward Ammonia by Nearly 100%. *Advanced Materials* **35**, 2306633 (2023).

36 He, W. *et al.* Splicing the active phases of copper/cobalt-based catalysts achieves high-rate tandem electroreduction of nitrate to ammonia. *Nature Communications* **13**, 1129 (2022).

37 Zhang, R. *et al.* Electrochemical nitrate reduction in acid enables high-efficiency ammonia synthesis and high-voltage pollutes-based fuel cells. *Nature Communications* **14**, 8036 (2023).
38 Li, P., Jin, Z., Fang, Z. & Yu, G. A single-site iron catalyst with preoccupied active centers that achieves selective ammonia electrosynthesis from nitrate. *Energy & environmental science* **14**, 3522-3531 (2021).
39 Yu, Y., Wang, C., Yu, Y., Wang, Y. & Zhang, B. Promoting selective electroreduction of nitrates to ammonia over electron-deficient Co modulated by rectifying Schottky contacts. *Science China Chemistry* **63**, 1469-1476 (2020).
40 Wu, Z.-Y. *et al.* Electrochemical ammonia synthesis via nitrate reduction on Fe single atom catalyst. *Nature communications* **12**, 2870 (2021).
41 Xu, Y. *et al.* Cooperativity of Cu and Pd active sites in CuPd aerogels enhances nitrate electroreduction to ammonia. *Chemical Communications* **57**, 7525-7528 (2021).
42 Ren, Y. *et al.* Microscopic-level insights into the mechanism of enhanced NH3 synthesis in plasma-enabled cascade N2 oxidation–electroreduction system. *Journal of the American Chemical Society* **144**, 10193-10200 (2022).
43 Álvarez-Constantino, A. M., Chaves-Pouso, A. & Fañanás-Mastral, M. Enantioselective Allylboration of Acetylene: A Versatile Tool for the Stereodivergent Synthesis of Natural Products. *Angewandte Chemie* **136**, e202407813 (2024).
44 Yin, H. *et al.* Alloying effect-induced electron polarization drives nitrate electroreduction to ammonia. *Chem Catalysis* **1**, 1088-1103 (2021).
45 Li, C. *et al.* Non-fullerene acceptors with branched side chains and improved molecular packing to exceed 18% efficiency in organic solar cells. *Nature Energy* **6**, 605-613 (2021).
46 Chen, D. *et al.* Synergistic modulation of local environment for electrochemical nitrate reduction via asymmetric vacancies and adjacent ion clusters. *Nano Energy* **98**, 107338 (2022).
47 Yin, D. *et al.* Synergistic active phases of transition metal oxide heterostructures for highly efficient ammonia electrosynthesis. *Advanced Functional Materials* **33**, 2303803 (2023).
48 Chen, F.-Y. *et al.* Efficient conversion of low-concentration nitrate sources into ammonia on a Ru-dispersed Cu nanowire electrocatalyst. *Nature nanotechnology* **17**, 759-767 (2022).
49 Lim, J. *et al.* Structure sensitivity of Pd facets for enhanced electrochemical nitrate reduction to ammonia. *ACS Catalysis* **11**, 7568-7577 (2021).
50 Li, J. *et al.* Efficient ammonia electrosynthesis from nitrate on strained ruthenium nanoclusters. *Journal of the american chemical society* **142**, 7036-7046 (2020).
51 Wang, K. *et al.* Intentional corrosion-induced reconstruction of defective NiFe layered double hydroxide boosts electrocatalytic nitrate reduction to ammonia. *Nature Water* **1**, 1068-1078 (2023).
