# Supplementary material for: Harnessing screw dislocations in shell-lattice metamaterials for efficient, stable electrocatalysts
Source: Nat Commun. 2025 Aug 7;16:7273. doi: 10.1038/s41467-025-62489-0 (PMC12332154; doi:10.1038/s41467-025-62489-0)
Supplement: Supplementary file 2 — Description of Additional Supplementary Files [file 41467_2025_62489_MOESM2_ESM.pdf]

Description of Additional Supplementary Files

Supplementary Movie 1. Mechanical response of FeCoNi dual-scale shell-lattice metamaterials and Ni foam.
